# Engineering Antibodies into Targeted Chimeras: From Recognition Modules to Programmable Degraders

**DOI:** 10.1002/advs.77142

**Published:** 2026-08-11

**Authors:** Kaige Chen, Quanyin Hu

**Affiliations:** ^1^ Pharmaceutical Sciences Division School of Pharmacy University of Wisconsin‐Madison Madison Wisconsin USA; ^2^ Carbone Cancer Center School of Medicine and Public Health University of Wisconsin‐Madison Madison Wisconsin USA; ^3^ Wisconsin Center for NanoBioSystems School of Pharmacy University of Wisconsin‐Madison Madison Wisconsin USA; ^4^ Lachman Institute for Pharmaceutical Development University of Wisconsin Madison Wisconsin USA

**Keywords:** antibody engineering, antibody‐associated targeted chimeras, programmable degraders, targeted protein degradation

## Abstract

Targeted chimeras have reshaped therapeutic design by converting target recognition into active degradation. As one of the most clinically established therapeutic modalities, antibodies provide high affinity and specificity to the protein of interest. Antibody‐associated targeted chimeras (AbTACs) are emerging as an important class of biologic degraders for targeted protein elimination, particularly of extracellular and membrane‐associated proteins. AbTACs offer a strategy to extend its recognition capacity beyond target binding by coupling defined degradation pathway. In these systems, antibody‐derived binders serve as programmable recognition modules, while conjugated ligands, encoded domains, recruited receptors, or nanoparticle scaffolds determine the target's intracellular fate. This review discusses the conceptual evolution of AbTACs and classifies current platforms according to scaffold architecture and engineering strategies, which link target binding to intracellular routing and subsequent clearance. Furthermore, it highlights the design principles and translational barriers that will guide the development of next‐generation antibody‐associated degraders.

## Graphical abstract

Antibody can be engineered into diverse lysosome‐targeting chimeras that convert target recognition into active degradation. By coupling antibody binding with recruited receptors, encoded domains, conjugated ligands, or nanoparticle scaffolds, these antibody‐associated targeted chimeras (AbTACs) platforms direct extracellular and membrane proteins toward intracellular clearance and support the development of next‐generation degraders.

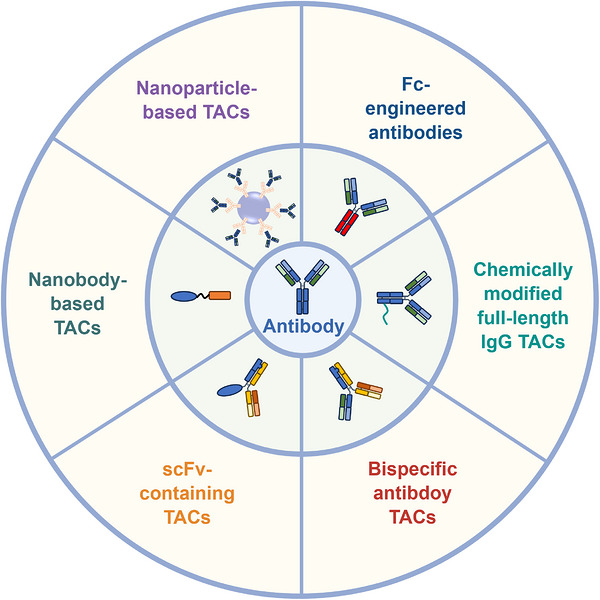

## 1 Introduction

Targeted chimeras (TACs) propose a class of engineered modalities designed to couple target recognition with high affinity to an active clearance process, which is referred to targeted protein degradation (TPD) [1, 2]. As the name suggests, TACs couple a target‐binding module with a degradation‐driving module, which converts the fate of the targeted protein of interest (POI). Once the POI is captured, the chimera re‐localizes it to an endogenous disposal pathway by recruiting effectors involved in trafficking, such as ubiquitin‐dependent degradation [3, 4], lysosomal trafficking [5], or receptor‐mediated clearance [6]. The design of TACs is not defined by a fixed structure but rather by a common logic in which the binding of POI is closely linked to active protein elimination.

This logic has been highly successful for intracellular proteins, especially through small‐molecule targeted protein degradation [7, 8, 9]. For early development of TACs of intracellular proteins, small molecules were applied as targeting modules for proteolysis targeting chimeras (PROTACs) to introduce POI to an E3 ligase and trigger degradation [10, 11]. In parallel, molecular glues offered a distinct degradative strategy by stabilizing interactions between a target protein and an E3 ligase, without the need for linker‐connected dual ligands [12]. However, the performance of small‐molecule TACs is highly constrained by their targeting modules. The function of small‐molecule targeting depends on well‐defined ligandable pockets, which make it difficult to engage extracellular, membrane, and protein–protein interface targets with sufficient affinity and selectivity. These targets are displayed on the cell surface, embedded in the plasma membrane, or released into the extracellular space, while their accessible surfaces are often broad and dynamic [13, 14, 15]. In the meantime, the degradation of small‐molecule TACs depends heavily on cytosolic access and on intracellular E3 ligases, which are poorly suited to directly handle extracellular targets.

Among the landscape of TACs, antibody‐associated TACs (AbTACs) have attracted interest in addressing these limitations [16, 17]. As one of the well‐studied and clinically applied drugs, antibodies were historically developed as highly selective therapeutic binders with extraordinary affinity [18, 19]. For decades, therapeutic antibodies were mainly used as blockers [20], neutralizers [21] or immune effector recruiters [22]. In these classical roles, binding itself was the endpoint of its final goal. As TPD developed, the focus shifted from whether the target can be bound to whether the binding can be coupled to a subsequent event, like an active target removal. Notably, this transition did not arise from a completely foreign concept, antibodies were already known to possess a similar logic as Fc receptor‐mediated endocytosis and intracellular trafficking can potentially contribute to the removal of bound antigens [23, 24]. This shift has repurposed the antibody from a static neutralizing agent into a programmable component of degrading systems.

In the design of AbTACs, the antibody serves as a recognition module, while the fate of the POI is determined by a module conjugated to, encoded in, or recruited to the antibody. A full‐length IgG can be converted into a degrader through chemical installation of a lysosome‐targeting ligand, polymeric module, or trafficking handle. The same recognition logic can be built into bispecific antibodies, which bind to multiple targets [25]. It would bridge a target with an internalizing receptor or membrane‐associated E3 ligase. The same recognition principle can be compressed into an IgG fragment‐derived format, such as a single‐chain variable fragment (scFv) [26, 27, 28] or a nanobody [29]. More recently, antibodies have been incorporated into nanoparticle‐supported systems that separate target recognition from the trafficking scaffold and allow modular assembly. The idea is simple across these diverse architectures: the antibody no longer only recognizes only the target; it also helps program the route by which the target is depleted. As a result, the emerging field of AbTACs has broadened to a diverse family of platforms, including lysosome‐trafficking chimeras, membrane E3 ligase‐recruiting degraders, Fc‐dependent clearance systems, signal‐directed sorting platforms, autophagy‐linked constructs, and nanoparticle‐enabled assemblies.

In this review, we will discuss the conceptual evolution, structural engineering, and mechanistic diversity of AbTACs. We first examined antibodies as programmable recognition modules and summarized the mechanistic features that distinguish antibody‐associated degradation from both traditional antibodies and classical small‐molecule TACs. Then we outlined the major trafficking routes and recruited effectors that have been coupled in current AbTAC designs. Further, we classified current platforms into several major categories, including conceptual precursors, multispecific antibodies, chemically modified full‐length antibodies, antibody fragment‐based systems, nanobody‐based constructs, and nanoparticle‐mediated platforms. Finally, we discussed the main translation challenges, including receptor availability, conjugation control, productive degradation versus simple internalization, tissue penetration, in vivo delivery, and safety. Taken together, these advances show that antibody‐based TACs do more than extend targeted protein degradation to a new set of targets. They redefine the antibody itself as a modular and programmable agent for active protein elimination.

## 2 Antibody‐Associated TACs as Programmable Degraders

### 2.1 Antibody as Targeting Module in TAC Design

In antibody‐associated TACs, antibodies are repurposed as programmable recognition modules that convert target binding into intracellular routing and degradation. Antibodies can be converted into TACs through several routes. As the most common therapeutic antibody class, Immunoglobulin G (IgG) is a Y‐shaped glycoprotein with a molecular weight of about 150 kD and accounts for more than 70% of total immunoglobulins in circulation [30, 31, 32]. It consists of two heavy chains and two light chains connected by disulfide bonds, which form two antigen‐binding regions (Fab) and one Fc region providing structural stability [33]. Due to its well‐defined architecture and multiple chemically accessible residues, IgG serves as a versatile scaffold for conjugation and development of AbTACs.

**FIGURE 1:**
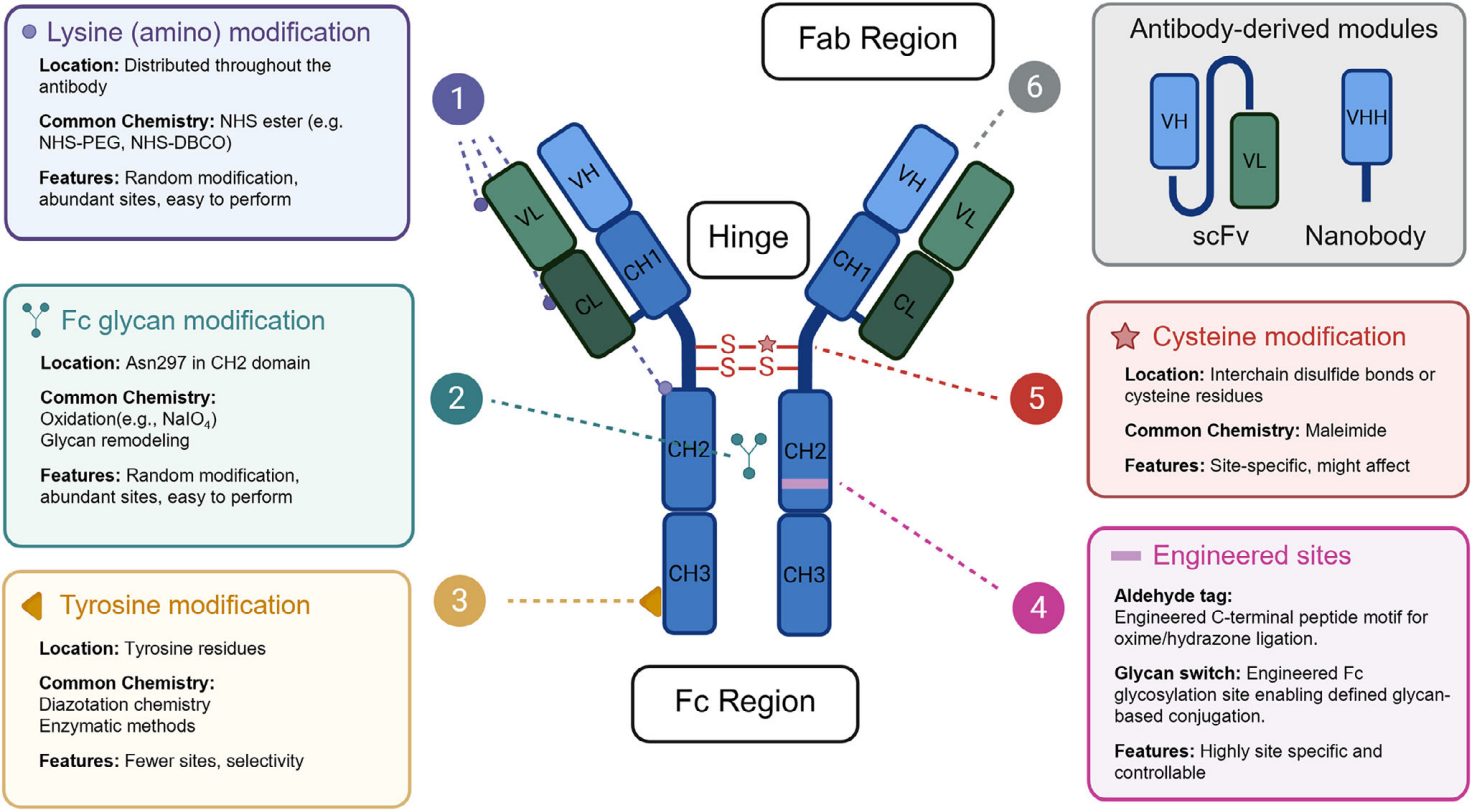
Schematic illustration of representative antibody modification sites used to construct antibody‐associated targeted chimeras. Figure created with BioRender.

A full‐length IgG can be chemically modified with a lysosome‐targeting ligand, such as M6P, GalNAc, folate, or other receptor‐binding modules at different sites (Figure 1). The most widely used strategy involves lysine residues bearing ε‐amino groups exposed on the IgG surface, which are conjugated via N‐hydroxysuccinimide (NHS) ester chemistry [34, 35, 36]. Although this approach was easy to execute and broadly applied to AbTACs, the large number and wide distribution of lysine residues led to heterogeneous products with uncontrolled conjugation [37]. In addition, AbTAC's design can also take advantage of the highly conserved N‐linked glycan in the Fc region [38]. As classically located at Asn297 in IgG, glycan modification provides a built‐in and structurally defined handle that is distinct from the broadly distributed lysine residues across the antibody surface. A common strategy is the periodate oxidation of terminal sugar residues to generate aldehyde groups, which can then undergo hydrazone ligation [39] oxime formation [40], or be further converted into bioorthogonal handles for subsequent click chemistry [41]. Compared to random lysine labeling, glycan‐centered modification offers a more defined route for ligand installation on full‐length IgG.

The following direction in antibody‐associated TAC design introduced site mutations to create defined chemical handles within the IgG scaffold. Instead of relying on natural residues, these strategies reprogrammed specific antibody regions for controlled conjugation. One approach involved installing engineered aldehyde‐bearing sites at selected positions, such as the hinge, CH1, or C‐terminus [42]. Another redesigned the Fc glycosylation site by shifting the conserved glycan from N297 to N298 through an Asparagine‐Asparagine‐Alanine‐Serine (NNAS) mutation, so that the engineered Fc glycan is highly sialylated, allowing subsequent oxidization and coupled by oxime chemistry [43]. Although interchain cysteines are another commonly used conjugation site in antibody‐drug conjugates via thiol‐reactive chemistries such as maleimide coupling [44], this strategy is less frequently adopted in antibody‐associated TAC systems. Disulfide reduction may compromise IgG structural stability and alter antibody geometry, thereby impairing efficient receptor engagement and ternary complex formation, both of which are essential for AbTAC.

Besides a full‐length format, IgG can also be engineered into a bispecific antibody, with one arm binding the target and the other recruiting a trafficking receptor or membrane‐associated E3 ligase. The same recognition logic can be compressed into antibody fragments such as scFv or nanobody for fully recombinant formats. This flexibility makes antibodies compatible with AbTACs [45], PROTABs [46], TransTACs [47], SignalTACs [48], and nanoparticle‐mounted degraders [49]. The antibody defines the target, while the conjugated module defines its fate.

### 2.2 Mechanistic Advantages of Antibody‐Associated TACs

**FIGURE 2:**
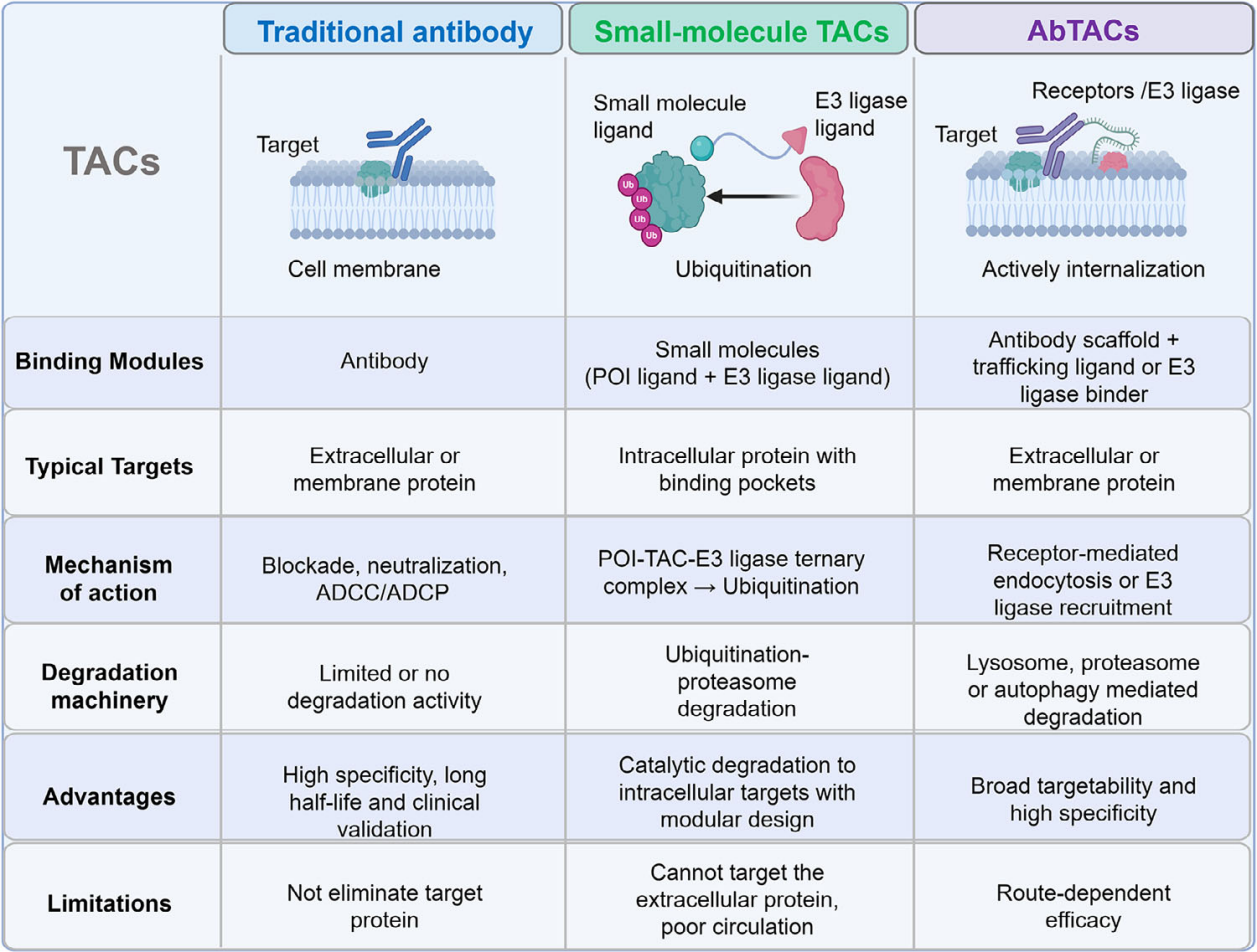
Conceptual comparison of traditional antibodies, small‐molecule TACs, and AbTACs. Figure created with BioRender.

Antibody‐associated TACs are mechanistically and pharmacologically distinct from classical small‐molecule TACs or traditional antibodies (Figure 2). Small‐molecule TACs usually rely on compact, intracellular ternary complex formation, whereas antibody‐associated TACs primarily act at the cell surface or in the extracellular space by coupling target recognition to receptor‐mediated routing or to membrane‐associated degradation machinery. They also differ from traditional antibodies, which mainly block signaling or neutralize ligands without necessarily removing the target protein itself. As a result, antibody‐associated TACs combine the binding selectivity of antibodies with an active mechanism for target depletion, giving them distinct mechanistic and pharmacological advantages.

The degradation efficiency of TACs is often impaired by the hook effect in ternary complex‐dependent systems. It occurs when an excess amount of TAC saturates the target protein and the recruited receptor or ligase separately, rather than promoting productive target–TAC–recruiter complex formation [50]. Small‐molecule TACs are particularly vulnerable to this problem because their target‐binding and recruiter‐binding groups are usually monovalent and depend on a narrow balance to form productive ternary complexes. At high concentration, these small molecules can independently occupy the POI and the LTR or the E3 ligase, thereby separating the two binding events rather than coupling them. Thus, the degradation efficiency showed a bell‐shaped decline at high concentration [51]. Compared with small‐molecule TACs, antibody‐associated TACs carry a lower risk of the hook effect, although this risk is not completely eliminated. Antibodies provide high‐affinity target engagement, slower dissociation, and multivalent binding [52]. These features can stabilize the interaction with the POI and increase local avidity at the cell surface. In an ASGPR‐engaging GalNAc‐LYTAC study, the authors did not observe an obvious hook effect within the tested concentration range, suggesting that productive ASGPR‐mediated ternary engagement was maintained under those conditions [42]. In the MONOTAB study, a nanoparticle‐based antibody‐associated degrader was directly compared with the bifunctional PD‐L1 degrader αPD‐L1‐GalNAc [49]. Although both showed similar activity at low concentrations, the bifunctional degrader exhibited a typical hook effect at higher concentrations, whereas the nanoparticle‐based format mediated more substantial PD‐L1 degradation without a hook effect.

The broad targetability of antibodies is powerful in LYTACs and related extracellular degraders. Many extracellular and membrane proteins lack deep pockets for small molecules. Their functional surfaces are often flat, glycosylated, flexible, or dominated by protein–protein interaction interfaces [53, 54]. Therefore, approximately 40% of the proteome remains difficult to target with small‐molecule TACs for degradation [55]. However, antibodies can recognize these surfaces through large and adaptable paratopes. They can bind folded extracellular domains with high affinity and selectivity, even when the target lacks a conventional targeting site. This is critical for extracellular targeted degradation, where the degrader must first capture a native protein on the cell surface or in the extracellular space. Although small molecules can work well when a defined binding pocket exists, antibodies can be generated against a much wider range of disease‐associated antigens. Antibodies can also target soluble extracellular proteins like cytokines and chemokines. For these targets, efficient target capture and subsequent receptor‐mediated clearance are the major determinants of activity due to their broad space distribution. While targeting membrane proteins, the distribution would be denser, and it requires productive surface engagement and co‐internalization toward degradation pathways.

### 2.3 Antibody‐Associated TACs Recruited Trafficking Routes/Receptors

**FIGURE 3:**
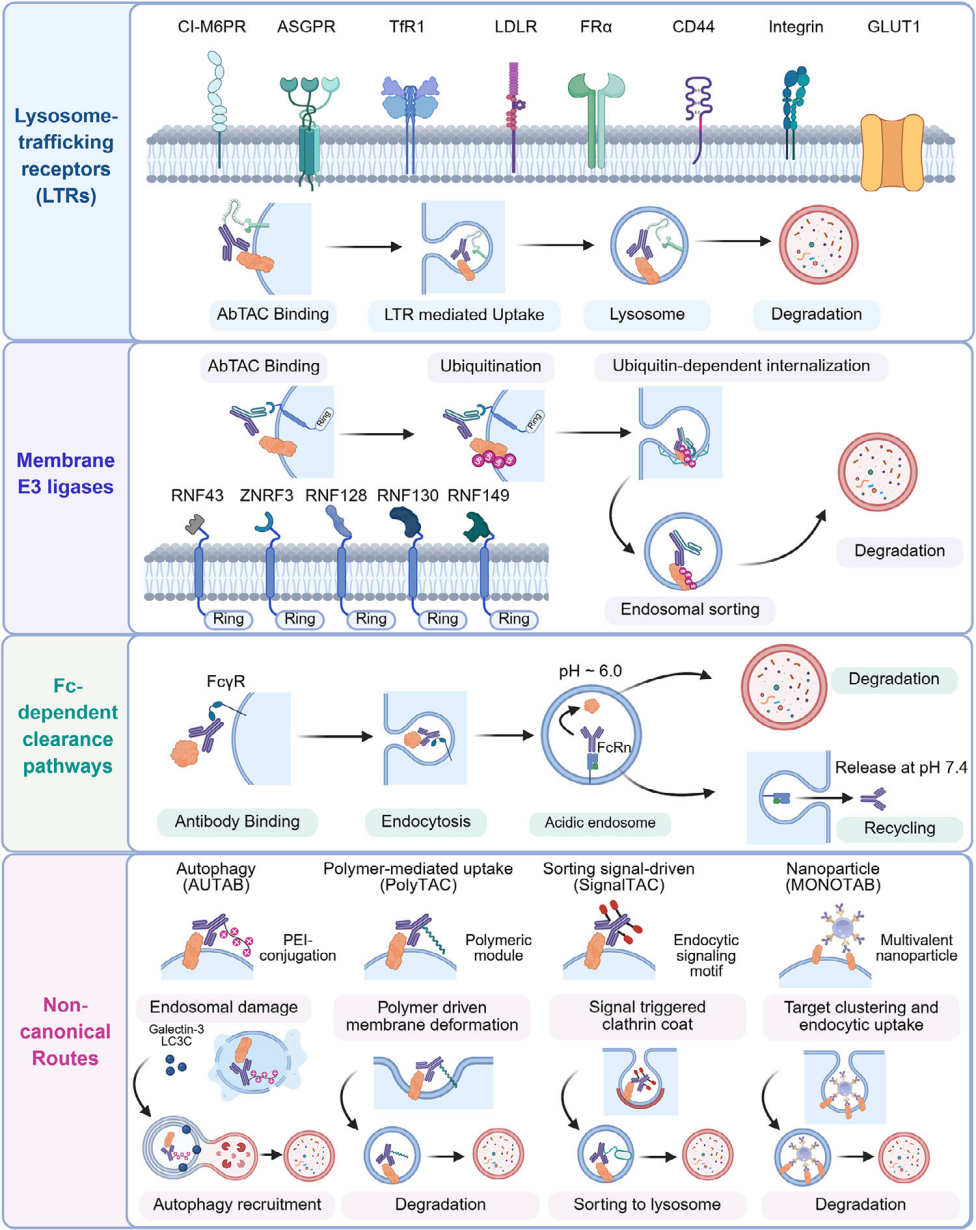
Overview of the major mechanistic classes of antibody‐associated TACs and their corresponding intracellular trafficking pathways leading to target degradation. Figure created with BioRender.

Compared to small‐molecule TACs, antibody‐associated TACs can access a much broader set of receptor systems for target routing. Most small‐molecule degraders are constrained by the availability of cell‐permeable ligands for both the POI and the recruited effector. This is especially limiting for extracellular and membrane proteins. Antibody‐associated TACs are more flexible because the antibody can provide target recognition, while the attached or recruited module can engage different internalization or degradation routes. As listed in Figure 3, antibodies have been coupled to ligands for lysosome‐trafficking receptors such as CI‐M6PR [56], ASGPR [42, 57], folate receptor [58, 59], TfR1 [47], LDLR [60], CD44 [61], Glut1 [62], and integrins [63] in the antibody‐associated TAC system. These receptors differ in tissue distribution, recycling behavior, uptake rate, and disease selectivity. For example, ASGPR supports hepatocyte‐selective degradation, folate receptor and CD44 can provide tumor‐associated uptake, and TfR1 or LDLR can exploit rapid endocytic recycling. In AbTAC [45] and PROTAB [46] formats, antibodies can also recruit membrane E3 ligases, including RNF43, ZNRF3, RNF128, RNF130, and RNF149, to promote ubiquitin‐dependent removal of membrane proteins. Other designs move beyond classical receptors by using Fc receptors, FcRn, autophagy‐related sensing pathways, or polymer‐driven membrane interactions.

## 3 Classification of Antibody‐Associated TAC Platforms

**FIGURE 4:**
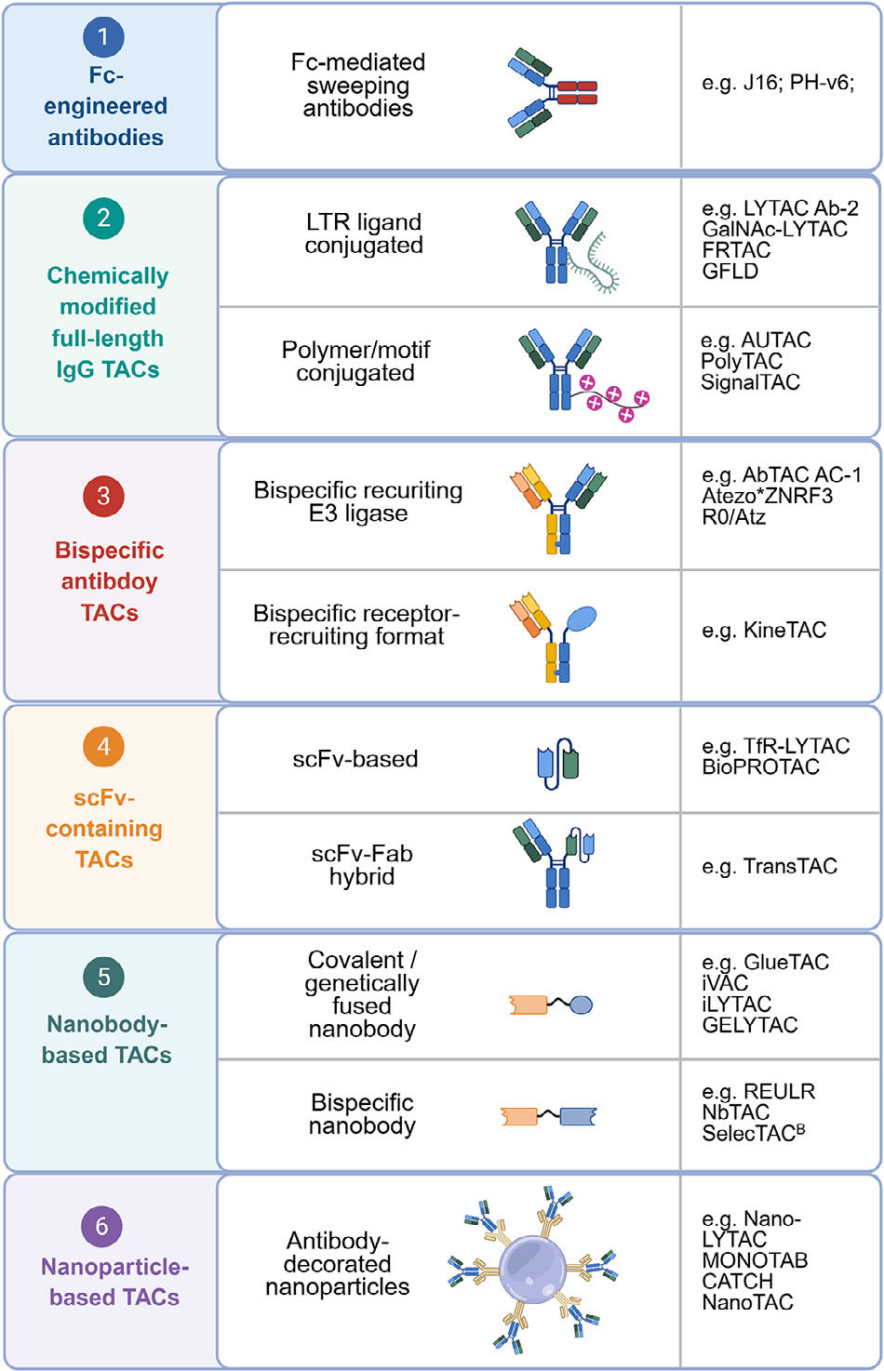
Classification of antibody‐associated TACs by scaffold architecture, conjugation strategy, and representative platform examples. Figure created with BioRender.

Given the rapid expansion of antibody‐associated TACs, a clear classification framework is needed before discussing individual platforms in detail. Although all antibody‐associated TACs link target recognition to protein depletion, they differ in scaffold format, construction strategy, recruited effector, and trafficking route. These differences shape how each system is built and how it works. Figure 4 summarizes the major classes discussed in the following section and highlights their representative structural logic.

### 3.1 Conceptual Precursors for Antibody‐Inspired Degraders

By coupling antigen recognition to Fc receptor‐mediated endocytic trafficking toward lysosomal degradation, natural IgG antibodies encode an AbTAC‐like logic. Although not typically classified as a canonical “degrader”, natural IgG antibodies can behave as degradation‐like trafficking that routes specific proteins toward endo‐lysosomal disposal with proper engineering. Dual recognition enables antibodies to function as target‐clearance agents for membrane or soluble targets via Fc‐FcR interactions, rather than through neutralization. However, the key limitation of conventional antibodies is that they are consumed upon binding to a membrane target. In the meantime, antigen accumulation would occur when the antigen binds to a soluble target [64], owing to the stabilizing effect of the antigen‐antibody complex.

In 2017, Clift et al. developed Trim‐Away, which is an antibody‐enabled strategy for the acute degradation of endogenous proteins [65]. This method delivers an antibody directly into the cytosol, where the antibody‐bound target is recognized by TRIM21, an endogenous Fc‐binding E3 ligase. After getting recognized and bound, TRIM21 then recruits the ubiquitin–proteasome system and drives rapid target destruction within minutes. The authors showed that Trim‐Away can deplete diverse unmodified proteins in mammalian cells, oocytes, and primary human cells without prior genome editing or target engineering. Although this work is not a canonical extracellular AbTAC platform, it established an important early principle that antibodies can function not only as binders, but also as active triggers of targeted protein degradation.

To address the existing problem of natural monoclonal antibodies, pH‐dependent antigen‐binding antibodies are designed to bind antigen at neutral pH but dissociate in acidic endosomes. By introducing histidine‐based engineering of the antigen‐antibody interface, the interface becomes protonated around pH 6, which weakens antibody binding and release. This feature would allow dissociated antigen to be transferred to the lysosome and eventually be degraded, while the antibody would recycle back to the plasma for another binding cycle. The earliest demonstration of this concept for antibodies was the engineered pH‐dependent antigen‐binding (“recycling antibody”) work by Tomoyuki Igawa and colleagues, showing that endosomal dissociation can promote lysosomal disposal of antigen, thereby freeing the antibody to return to circulation via FcRn and diverting it from lysosomal destruction [66]. Recycled antibodies go back to the cell surface for release at neutral pH, enabling iterative antigen capture and degradation. Another illustration comes from pH‐sensitive anti‐PCSK9 engineering, where Chaparro‐Riggers applied a histidine‐scanning mutagenesis to generate a pH‐switch. This provides a clear mechanistic demonstration that pH‐switched Fab binding in IgG can reprogram endosomal sorting and decouple antigen and target disposal from antibody salvage, which underlies recycling and sweeping TAC designs [67]. This concept was later extended to toxin clearance. In 2019, engineered pH‐dependent anti‐SEB antibodies were shown to accelerate elimination of Staphylococcal enterotoxin B in mice while maintaining neutralizing activity [68]. The study further linked stronger pH dependency to lower antigen exposure in vivo, supporting the catch‐and‐release mechanism.

Sweeping antibodies were introduced as an explicit attempt to create an IgG‐mimic ligand that sweeps endocytic receptors, continuously removing soluble ligands with an uptake accelerator. Fc engineering then biases the fate of the antibody and the antigen in opposite directions. It demonstrated that increasing Fc binding to mouse FcγRII and FcγRIII markedly accelerated antigen clearance, and that a human IgG1 Fc variant engineered for selectively higher binding to human FcγRIIb accelerated soluble antigen clearance in human FcγRIIb‐transgenic mice without shortening antibody half‐life [69]. To boost clearance efficiency, Hori developed a novel Fc variant with enhancing FcγRIIb engagement and FcRn binding. In cynomolgus monkeys, this integrated design enables active removal of the extracellular target in circulation while maintaining pharmacokinetics close to a wild‐type IgG1 baseline [70].

A related conceptual precursor emerged from antibody–enzyme conjugates designed for targeted glycan removal rather than canonical protein degradation [71]. In this strategy, trastuzumab was linked to a sialidase to selectively erase tumor‐associated sialoglycans from HER2‐positive cancer cells. Although the degraded entity was not a single protein target, the work introduced an important principle. Antibody recognition was coupled to a localized catalytic event that actively removed a disease‐associated surface component after binding. This shifted the antibody from a simple blocking agent toward a programmed effector of target clearance. In this sense, the study did not establish a classical AbTAC platform, but it broadened the conceptual boundary of antibody‐guided degradation by showing that antibodies could direct selective catalytic removal of complex extracellular structures.

### 3.2 Multi‐Specific Antibody as AbTAC

The IgG‐derived antibody scaffold is inherently modular and provides two antigen‐binding arms in the Fab region, which can be designed for distinct specificities. Instead of using both Fab arms to recognize the same antigen, binding specificity can be engineered to recognize different targets. This dual‐binding architecture allows the bispecific to physically bridge two proteins and force a closing interaction on the cell membrane. Functionally, that proximity is the key event: once the target protein is coupled to an internalizing receptor or to a membrane‐associated degradation machinery component, the complex can be routed into endocytic and lysosomal pathways, resulting in depletion of the target rather than simple blockade. For this reason, bispecific antibodies are well‐suited to serve as TACs. In this case, the bispecific antibody provides both the recognition logic and the spatial organization required for targeted degradation of cell‐surface proteins.

In 2021, Cotton et al. developed a fully recombinant bispecific antibody to function as an antibody‐based PROTAC [45]. Two half‐IgG arms are expressed separately and assembled in vitro to form a bispecific antibody via point mutation. With one arm binding to the target protein and another arm binding to recruit the membrane‐bound E3 ligase, this bispecific antibody bridges them and forms a ternary complex. This forced proximity facilitates the intracellular ubiquitination of the target protein's cytoplasmic tail, triggering its rapid internalization and subsequent lysosomal degradation. By applying PD‐L1 as the model target and RNF43 as the cell‐surface E3 ligase, they demonstrated that a bispecific antibody induced substantial PD‐L1 loss (up to 63%) in multiple cancer cell lines. Instead of relying on a small‐molecule ligand for a cytosolic target in the early version of LYTAC (2020 Nature), this work expands the paradigm of TACs with a genetically encoded protein format without involvement of any small‐molecule ligands.

**FIGURE 5:**
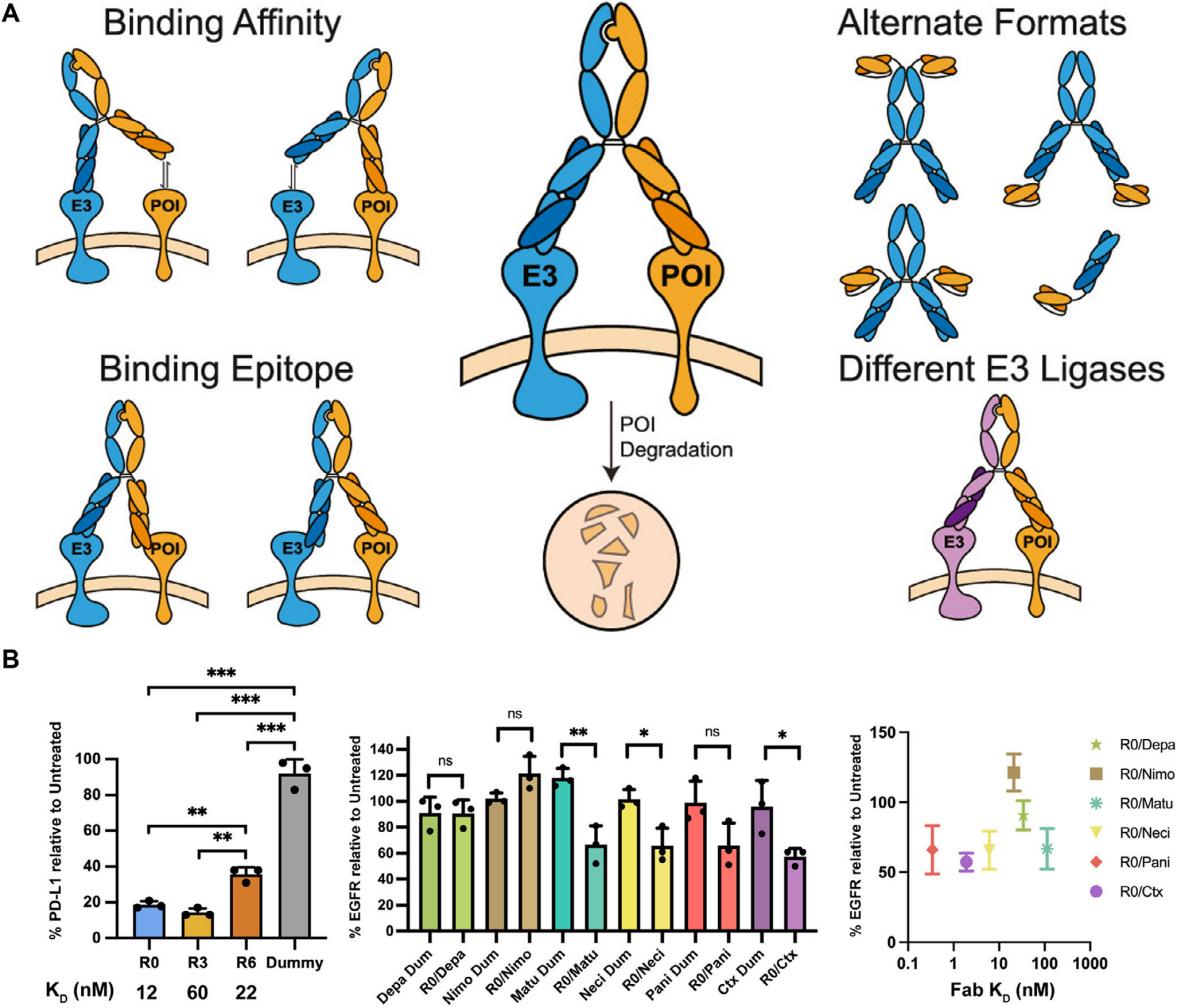
(A) Schematic of AbTACs format. (B) AbTAC optimization of productive degradation with various epitope geometry and scaffold architecture. Adapted with permission from Ref. [72]. Copyright 2022, American Chemical Society.

Building on their first proof of concept, the same team provided a systematic roadmap for optimizing the degradation performance of the AbTAC platform [72]. To determine the key contributor to degradation, the authors varied both the bispecific antibody and the target protein to compare different epitopes on the recruited transmembrane E3 ligase and the target protein (Figure 5A). They found that, rather than affinity, binding epitopes are critical to form a productive degradation complex, which requires a suitable spatial arrangement to trigger degradation (Figure 5B). Similar results were observed in both RNF43 and ZNRF3 AbTACs. They showed that AbTACs can be improved by tuning IgG scaffold flexibility, valency, and arm orientation, thereby establishing a framework for optimizing cell‐surface protein degradation. This work transitions the AbTAC technology from concept to a rationally engineered framework, offering a highly programmable optimization paradigm for developing biologic degraders as bispecific antibodies.

A similar strategy has been introduced as a bispecific antibody platform for degrading cell‐surface receptors by Marei et al. called proteolysis‐targeting antibodies (PROTABs) [46]. They achieved a critical milestone by translating this bispecific degradation concept into a successful in vivo application. They engineered PROTABs to bridge disease‐driving receptors, such as IGF1R, with the transmembrane E3 ligase ZNRF3. While targets like IGF1R are widely expressed on healthy cells, the recruited ZNRF3 exhibits a highly restricted expression profile, which is heavily enriched in specific tumor microenvironments (such as colorectal cancer) but largely absent in normal tissues. Unlike traditional inhibitors or antibodies, this design offers selectivity, allowing tissue‐restricted ligase expression to make receptor degradation more selective than receptor blockade alone. As a result, the bispecific feature of PROTAB acts like a tumor‐specific switch. PROTABs can be extended across ligases and targets, and define practical rules for engineering potent, tissue‐selective surface degraders.

Rather than restricting to membrane E3 ligases, Zhang et al. introduced a different strategy involving a distinct pathway for transferrin receptor (TfR) transfer [47, 73]. A heterobispecific antibody was designed to bind POI with one arm, whereas another arm engages TfR1, a receptor with rapid constitutive endocytosis and elevated expression in many cancer cells and activated T cells. Bypassing the ubiquitin system, TransTACs simultaneously physically drags the target protein into a rapid endocytic cycle with TfR1. Unlike the structure of a traditional bispecific antibody, TranTACs are engineered constructs prepared by integrating a bivalent architecture of scFv joined to an anti‐POI scFv, genetically fused to the Fc domain via a “Knobs‐into‐Holes” mutation. However, the rapid recycling behavior of TfR1 also impairs degradation efficiency. The authors addressed this by reducing recycling with a cathepsin‐cleavable linker that enabled separation of the target from TfR1 in endosomes, thereby allowing TfR1 to recycle while routing the target to lysosomal degradation. With this design, they achieved efficient degradation of structurally distinct membrane proteins, including CAR, PD‐L1, EGFR, and CD20, often exceeding 80%.

**FIGURE 6:**
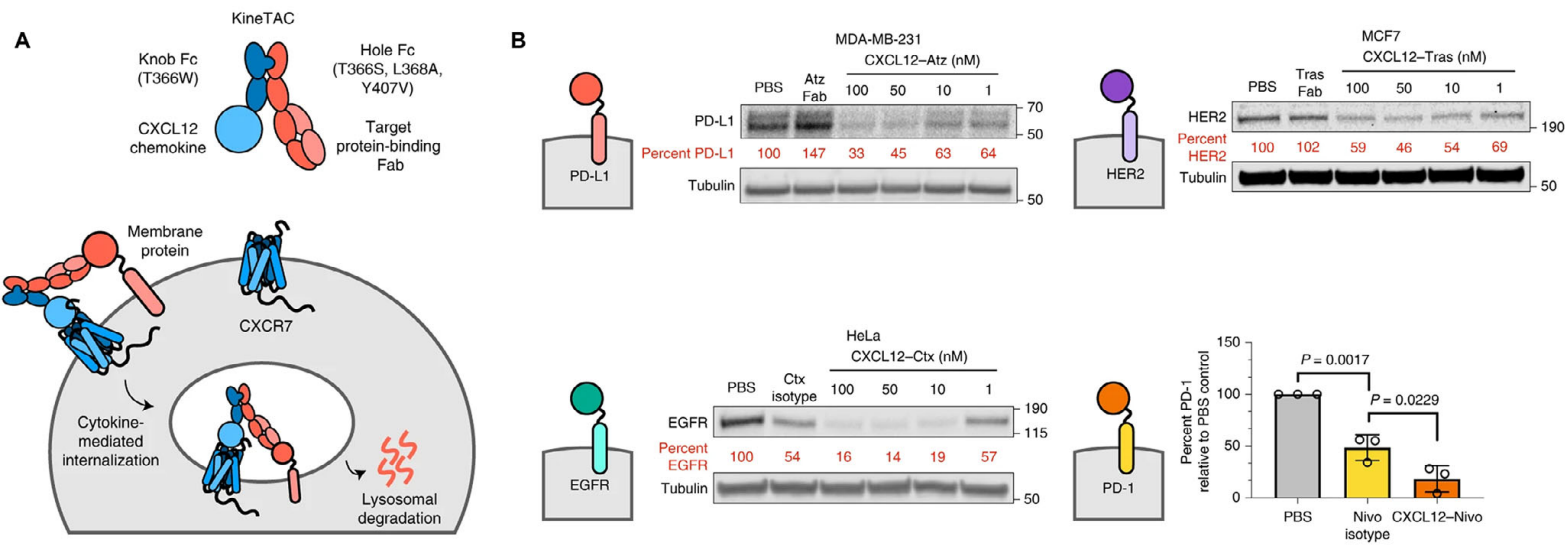
(A) Schematic of KineTAC format. (B) The degradation of different POI depending on the change of target protein binding Fab to PD‐L1, HER2, EGFR, and PD‐1. Adapted under the terms of the Creative Commons Attribution 4.0 International License Ref. [74] Copyright 2022, The Authors, published by Springer Nature.

Fused protein targeting internalizing receptors, and the target protein can also work as a bispecific TACs system. KineTACs are a fully recombinant bispecific degradation platform for cell‐surface and extracellular proteins [74]. One arm contains a cytokine, such as CXCL12, to recruit an internalizing cytokine receptor, while the other arm binds the target protein (Figure 6A). This design converts receptor‐mediated uptake into lysosomal delivery and target depletion. In the study, CXCL12‐based KineTACs degraded multiple membrane proteins, including PD‐L1, HER2, EGFR, CDCP1, TROP2, and PD‐1, and also promoted the uptake of soluble VEGF and TNF‐α (Figure 6B). The platform is modular, genetically encoded with “Knob‐into‐holes” for assembly, and does not require chemical conjugation, which makes it easier to build and optimize.

Taken together, these studies show the reason why bispecific antibodies are well‐suited for extracellular targeted degradation. Their value lies not only in dual recognition, but also in the ability to impose a defined spatial relationship between a target protein and a chosen cellular effector. This multispecific antibody turns binding into routing, and routing into depletion via biological design. Across AbTACs, PROTABs, and TransTACs, the bispecific antibody format remains the common design logic. It provides a fully recombinant framework for engineering membrane protein loss. In this sense, bispecific antibodies are not simply carriers of degradation modules. They are the organizing scaffold of the degrader itself.

### 3.3 Modified Full‐Length Antibody as TACs

As described previously, a full‐length IgG antibody consists of two parts: the Fab region, which determines the binding specificity, and the Fc region, which determines what happens after binding. The engineered modification of the Fc region endows the antibody with new functionality and can potentially reshape biological behavior, such as acting as a TAC. The pioneering first generation of LYTAC was established by Banik as a general strategy for degrading extracellular and membrane‐associated proteins through the lysosomal pathway [75]. The first generation of LYTAC was reprogrammed from existing antibodies by chemically conjugating a multivalent CI‐M6PR‐binding glycopolypeptide, shuttling the POI to the ubiquitous lysosome. In the original CI‐M6PR LYTAC design, the M6Pn glycopolypeptide was conjugated to lysine residues of full‐length antibody through bicyclononyne–N‐hydroxysuccinimide (BCN‐NHS) labeling followed by copper‐free azide–alkyne click conjugation. The study first validated this concept with antibody‐mediated uptake of soluble proteins, then extended it to therapeutically relevant membrane targets. Cetuximab‐based LYTACs induced more than 70% EGFR degradation, and an atezolizumab‐derived LYTAC achieved about 70% PD‐L1 degradation in a CI‐M6PR high‐expression lymphoma cell line. These works provided the first modular route for directing extracellular proteins to lysosomes by a modified antibody as a targeting module. By doing so, it expanded targeted degradation to a large class of proteins beyond the reach of classical intracellular PROTAC logic. The behavior of a full‐length antibody as a modular recognition element can therefore be shifted by chemical modification of a lysosome‐targeting ligand, converting an occupancy‐based binder into a degradation‐inducing construct. However, one point that may need to be stated more precisely is that the first‐generation LYTACs involved random lysine‐based conjugation across the antibody scaffold, rather than a defined modification of a certain region in the antibody.

A later study addressed two early limitations of the first LYTAC design [42]. One was the broad tissue distribution of CI‐M6PR. The other was the structural heterogeneity introduced by random conjugation. To overcome these issues, the authors turned to the liver‐restricted asialoglycoprotein receptor (ASGPR) and developed GalNAc‐LYTACs based on full‐length antibody scaffolds. In this design, a tri‐GalNAc ligand was attached to the antibody to route the target bound through ASGPR‐mediated uptake. The later part of the study then moved beyond nonspecific lysine labeling and introduced site‐specific conjugation on defined positions of the antibody scaffold. This provided more chemically defined constructs and allowed the relationship between the conjugation site, degradation activity, and in vivo behavior to be examined more clearly. The significance of this work was therefore not only tissue selectivity. It also showed that antibody‐based extracellular degraders could shift from heterogeneous chemical conjugates toward more controlled molecular formats.

Mukai et al. introduced another enzyme‐free strategy for site‐specific conjugation by engineering an Fc glycosylation switch in an antibody [43]. They introduced the NNAS Fc mutation, which shifted the native glycosylation site and produced highly sialylated Fc glycans during normal expression. These glycans could then be selectively oxidized and coupled to a synthetic high‐affinity bisM6P ligand through simple oxime chemistry. In this way, the antibody was converted into a more defined M6PR‐engaging degrader without requiring exogenous enzymatic processing. The authors also used an Fc‐silenced format, which reduced native effector activity and made the scaffold more suitable for degradation‐driven applications. Functionally, the platform extended M6PR‐based degradation beyond membrane proteins to soluble inflammatory targets. BisM6P‐conjugated anti‐TNF efficiently internalized TNF into endolysosomal compartments and depleted TNF from culture media in both cancer cell lines and human immune cells. The same principle was also applied to serum amyloid A. From an antibody engineering perspective, the significance of this work lies in a simple point. Fc redesign was used not only to control conjugation, but also to expand the usable target space of full‐length antibody‐associated LYTACs.

A later study further refined the CI‐M6PR LYTAC platform by replacing the original heterogeneous M6Pn glycopolypeptides with structurally defined M6Pn peptides conjugated to full‐length antibodies [56]. This work did not introduce a new receptor but instead clarified why CI‐M6PR‐mediated degradation of membrane proteins is often incomplete. Genome‐wide screening revealed that retromer‐dependent recycling can return LYTAC–CI‐M6PR complexes to the cell surface before productive degradation, and that endogenous M6P‐modified ligands also compete for receptor occupancy. These findings shifted the field from simply building LYTACs to understanding how receptor recycling, ligand competition, and endolysosomal trafficking set the ceiling for degradation efficacy.

**FIGURE 7:**
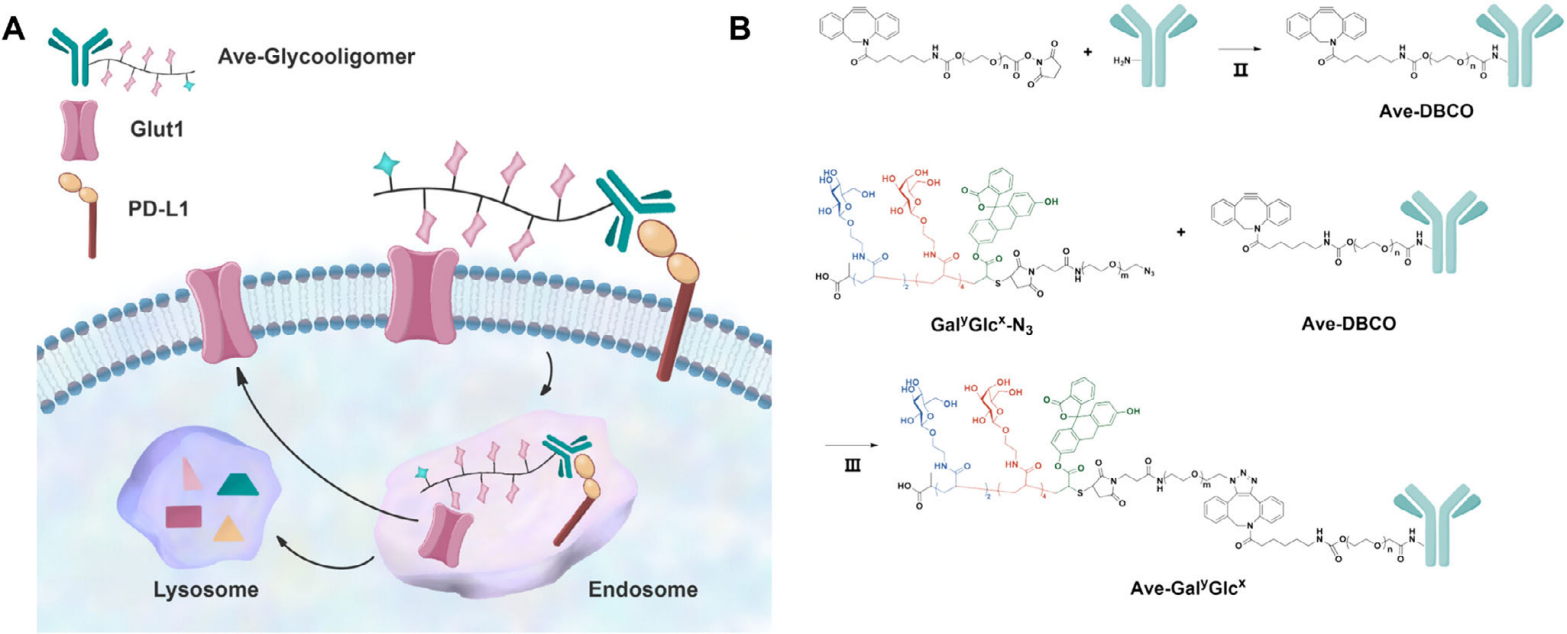
(A) Schematic of GFLD format. (B) Syntheses route of Ave−Glycooligomer conjugates. Adapted with permission from Ref. [62]. Copyright 2024, American Chemical Society.

Instead of traditional LTR and E3 ligase, Luo et al. expanded the rational design of endogenous internalization machinery and introduced Glut1‐Facilitated Lysosomal Degradation (GFLD) in their study [62]. They converted anti‐PD‐L1 antibody avelumab into a Glut1‐directed lysosomal degrader by conjugating glycooligomer ligands to the antibody through a two‐step bioorthogonal route (Figure 7A). They synthesized antibody–glycooligomer conjugates by attaching azide‐functionalized Glut1‐binding glycooligomers to dibenzocyclooctyne (DBCO)‐modified avelumab through CLICK reaction (Figure 7B). GFLD takes a different route to selective membrane protein degradation. Instead of relying on a broadly expressed lysosome‐shuttling receptor, GFLD uses the key nutrient transporter Glut1, which is often elevated in tumor cells with high glucose demand. That choice matters because it ties degrader activity to a metabolic feature of the target cell. In their design, selectivity does not come from the antibody arm by itself. It also comes from the receptor that drives uptake. The value of the platform, therefore, lies in this design logic, which links membrane protein depletion to a transporter already favored by tumor cells.

**FIGURE 8:**
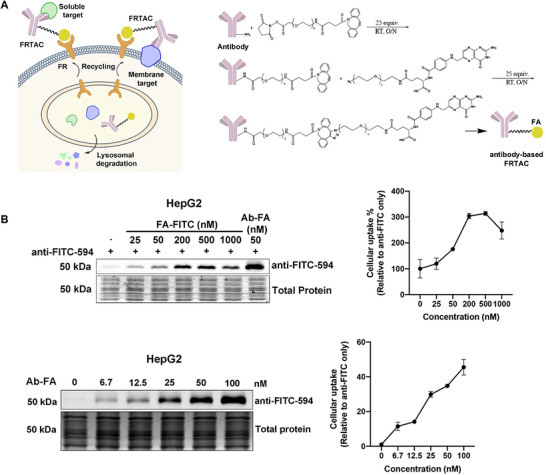
(A) Schematic of FRTAC‐induced lysosomal targeted protein degradation. (B) Uptake of anti‐FITC‐594 50 nM) in HepG2 cells treated with Ab‐FA (25 nM) and FA‐FITC (200 nM). Adapted under the terms of the Creative Commons Attribution 4.0 International License. Ref. [58] Copyright 2024, The Authors, published by Springer Nature.

A related line of work extended this idea further by conjugating folate to an antibody to form a tumor‐selective LYTAC. Unlike Glut1, which contributes to selectivity through the metabolic state of tumor cells, folate receptors serve as a more direct tumor‐associated surface lysosome‐trafficking receptor. Folate receptor expression is elevated in many cancers, whereas it remains limited or poorly accessible in most normal tissues. Folate receptors undergo efficient receptor‐mediated endocytosis, which makes it well suited to carry antibody‐bound targets into the lysosomal pathway. A practical antibody‐associated strategy using folate receptors has been developed by Zhou et al. [58]. Existing antibodies were converted into degraders by sequential chemical modification in a two‐step format. The antibody was first labeled with DBCO‐PEG3‐NHS on lysine residues and then coupled to azido‐PEG‐folate by click chemistry (Figure 8A). To show the advantage of antibody‐associated TACs over small‐molecule‐based TACs, the authors compared an antibody‐based FRTAC with a smaller folate‐linked analog, FA‐FITC. The antibody format resulted in greater uptake of the model protein cargo. At 50 nM Ab‐FA, internalization of anti‐FITC‐594 was greater than that achieved with 200 nM FA‐FITC. The smaller degrader showed a clear hook effect at high concentration, whereas the antibody‐associated format performed more steadily (Figure 8B). In this setting, the advantage of the full‐length antibody was not only binding. This comparison proves that for internalizing bulky extracellular proteins, the macromolecular antibody‐associated TACs fundamentally outperform traditional small‐molecule logic. The same design was then transferred to therapeutic antibodies. With cetuximab as the binder, the optimized Ctx‐FA bearing a 2 kDa PEG linker showed potent EGFR degradation, with DC50 values of 0.41 nM in Fadu cells and 0.24 nM in HeLa cells, and Dmax values of about 75%–80%. EGFR loss became apparent after 4–6 h and persisted for at least 72 h. The platform was further extended to atezolizumab and an anti‐CD47 antibody, giving Atz‐FA and Ab2‐FA, which degraded PD‐L1 and CD47 in cancer cells.

Further expanding the folate receptor‐mediated degradation paradigm, Xiao et al. introduced polyvalent folate receptor‐targeting chimeras in 2025 [59]. Knowing that monovalent ligand recruitment is limited by endocytic kinetics, their study achieved high‐density crosslinking by conjugating a multivalent folate polymer to the antibody. The authors showed that degradation through FR depended strongly on how folate was displayed on the antibody scaffold. FR‐Ctx with a folate–antibody ratio of 15 almost completely reduced EGFR at 10 nM, whereas FR‐Ctx with FAR 2 showed little effect at the same dose. This difference was linked to receptor organization at the membrane. High‐FAR constructs bound FRα much more strongly and induced visible FRα microclusters on the cell surface. At the same time, binding to EGFR or PD‐L1 was retained. Functionally, this design pushed degradation into the subnanomolar range. FR‐Ctx showed a DC50 of 0.099 nM for EGFR, and FR‐Atz showed a DC50 of 0.080 nM for PD‐L1, with Dmax values above 80%. This work therefore developed the folate receptor strategy from a workable antibody conjugation to a more engineered full‐length antibody platform, in which conjugation density itself became a determinant of receptor clustering, uptake efficiency, and degradative output.

With a similar logic of hijacking tumor‐associated surface adhesion molecules, Zhou et al. developed integrin‐targeting chimeras (ITACs) as a cancer‐selective platform for the degradation of both soluble and membrane‐bound proteins [63]. To construct ITACs, full‐length antibodies were chemically conjugated to a cyclic RGD peptide, a high‐affinity ligand for integrins overexpressed in many tumors. Antibodies such as cetuximab were first modified with DBCO‐PEG‐NHS and then coupled to azide‐functionalized cRGD by click chemistry. A key insight of this work is the critical role of structural flexibility for degradation with membrane target accomplished by linker length. The internalization of soluble targets like NA‐650 was barely affected by linker, while it is highly enhanced with longer PEG linkers involving membrane‐bound EGFR target. This result suggests that spatial flexibility is more important when the antibody‐associated LYTACs must engage both integrin and a membrane‐bound target on the cell surface.

Cheng et al. developed an antibody degrader that does not depend on a second membrane receptor but instead triggers cellular autophagy, termed autophagy‐inducing antibodies (AUTABs) [76]. To construct AUTABs, the researchers chemically conjugated targeting antibodies with linear polyethylenimine (PEI), a simple cationic polymer that interacts strongly with negatively charged membrane lipids. Once the AUTAB binds its specific receptor on the plasma membrane, the complex undergoes receptor‐mediated endocytosis. Then, the conjugated PEI damages the endosomal membrane and is sensed by damage sensor galectin‐3, subsequently recruiting LC3C‐dependent autophagy. Consequently, it drives the internalized receptor toward lysosomal degradation. The efficiency of this strategy has been verified with commercial antibodies targeting PD‐L1, EGFR, and CD73. Because AUTABs initiate degradation through the interaction between the charged polymer and the cell membrane, they do not rely on the expression levels of specific shuttling receptors, which makes the platform easier to apply across different targets and cell types. AUTABs highlight a distinct feature of full‐length antibody modification as TACs that chemical modification can impart entirely new intracellular routing mechanisms onto classical antibodies.

A related step was reported by Lu et al., who showed that synthetic polymers can also redirect intracellular trafficking, thereby reducing the need for a specific LTR [77]. Without involving LTRs, polymeric lysosome‐targeting chimeras (PolyTACs) directly reacted with endogenous thiol groups overexpressed on tumor cell surface. In their main construct, atezolizumab was conjugated to a thiol‐reactive polymer containing about 10 pyridyl disulfide units and about 40 hydrophilic repeat units. The polymer carried a terminal tetrazine, whereas the antibody was modified on lysine residues with trans‐cyclooctene (TCO). Conjugation was then completed via tetrazine–TCO click chemistry, yielding an average of approximately 12 polymer chains per antibody. The pyridyl disulfide in the polymer engages thiol groups on the membrane, forming a multivalent interaction that subsequently leads to local membrane deformation. Driven entirely by this physical deformation, the cell initiates a non‐classical endocytic pathway, mechanically dragging the tightly cross‐linked PolyTAC‐target complex directly into the lysosome for degradation. By replacing a defined shuttling receptor with polymer‐driven membrane interactions, PolyTACs offer a more programmable route for degrading difficult extracellular targets.

**FIGURE 9:**
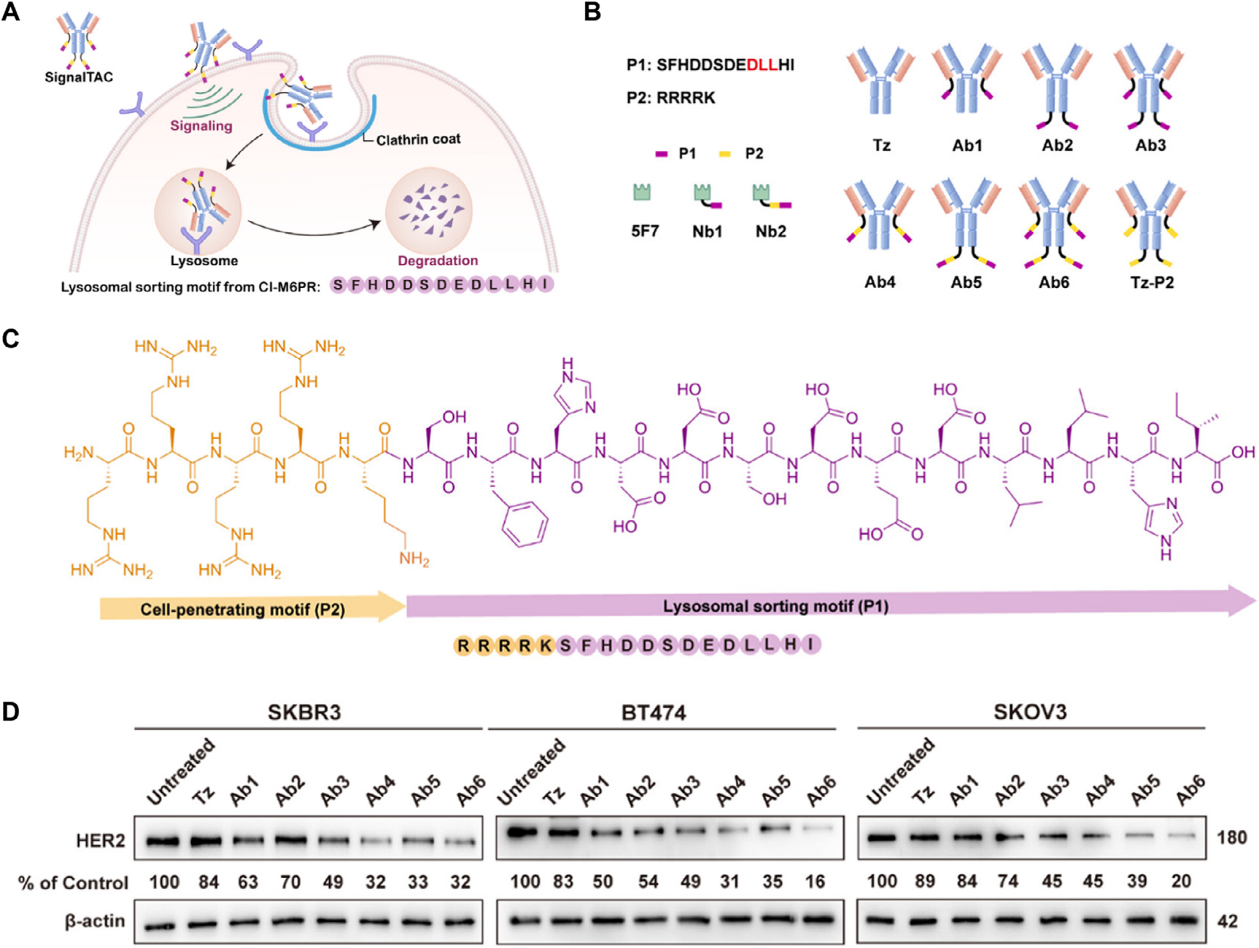
(A) Schematic of concept of SignalTAC. (B) Construction of SignalTACs based on a nanobody (5F7) or a humanized IgG monoclonal antibody (Tz). (C) Structure of P1 and P2. (D) Western blot analysis of HER2 in SKBR3, BT474, and SKOV3 cells after treatment with 100 nM Tz or Tz‐based SignalTACs. Adapted with permission from Ref. [48]. Copyright 2023, American Chemical Society.

Yu et al. introduced SignalTACs as a different solution for extracellular protein degradation [48]. Instead of relying on a cell‐surface shuttling receptor, this platform used an intracellular sorting signal to redirect the bound target into the endolysosomal pathway (Figure 9A). The core design was a short dileucine‐based motif derived from the cytosolic tail of CI‐M6PR. This motif was genetically fused to the target binder, together with a short cell‐penetrating peptide, thereby enabling access to clathrin‐dependent internalization routes without requiring a dedicated surface receptor (Figure 9B,C). A notable feature of the platform was its fully encoded architecture. The same design could be built on full‐length antibodies, nanobodies, or peptides without chemical conjugation. In the antibody setting, tetravalent formats with sorting modules on both heavy and light chains performed better than divalent versions, indicating that construct geometry still mattered even in this receptor‐independent design (Figure 9D). SignalTACs also showed broad target scope. They degraded not only conventional membrane proteins, but also more difficult targets such as CD20, which internalizes poorly, and CD71, which is usually biased toward recycling. The significance of this work lies in its change in effector logic. It shifted extracellular targeted degradation from receptor recruitment to signal‐directed trafficking.

### 3.4 Antibody Fragment‐Based TACs

As a compact antibody fragment composed of the VH and VL domains connected by a flexible peptide linker, scFv preserves the antigen‐binding specificity of a conventional antibody but removes the Fc region [27, 28]. Thus, it makes scFv well‐suited for a modular targeting module in antibody‐associated TACs, especially when the binder must be directly linked to a fusion protein rather than installed through chemical modification of a full‐length IgG. In antibody‐associated TACs, this feature has led to two related but distinct designs. One uses scFv as the extracellular targeting arm in a genetically encoded lysosomal degrader, while the other uses scFv as an intracellular targeting fused to an E3 ligase domain.

A representative scFv‐based extracellular degrader was reported by Su et al. in the OMV‐LYTAC study [78]. The authors first constructed a genetically encoded TfR‐LYTAC by fusing the avelumab scFv to a transferrin receptor‐binding peptide via a flexible linker. The scFv preserved PD‐L1 recognition, while the TfR‐binding peptide recruited the complex into TfR‐mediated endocytosis. In cultured cells, this fusion protein bound both PD‐L1 and TfR, formed a ternary complex, and eventually drove PD‐L1 transport to LAMP1‐positive lysosomes. PD‐L1 depletion was both dose‐ and time‐dependent and was blocked by a free TfR‐binding peptide, supporting a TfR‐dependent lysosomal mechanism. The main limitation appeared in vivo applications. Soluble TfR‐LYTAC showed poor tumor retention and failed to reduce PD‐L1 in solid tumors. The authors therefore displayed TfR‐LYTAC on bacterial outer membrane vesicles (OMVs) by fusing it to an OMV surface protein, ClyA. Furthermore, an MMP2‐cleavable PLGLAG linker was inserted between ClyA and TfR‐LYTAC, granting release of the degrader in the tumor microenvironment. This design changed the role of scFv‐based TACs from a simple recombinant fusion protein into a delivery‐coupled biologic degrader. OMV‐LYTAC prolonged circulation, improved tumor accumulation and reduced tumor PD‐L1 level. It showed a stronger antitumor activity than soluble TfR‐LYTAC. This work shows that scFv can serve as a compact targeting unit, while the delivery platform determines whether the degrader can function in vivo.

Extending scFv‐based TACs from extracellular trafficking to intracellular protein degradation, Chisholm et al. developed a BioPROTAC for selective degradation of misfolded SOD1 [79]. The study focused on the misfolded protein superoxide dismutase 1 (SOD1) in amyotrophic lateral sclerosis, in which precise selectivity would be challenging. As natural SOD1 remains functionally important, whereas misfolded SOD1 variants form toxic aggregates. Thus, a suitable degrader should distinguish a pathological conformation from the correctly folded protein. To achieve this, the authors converted misfolded SOD1‐specific antibodies into scFv intrabodies and fused them to E3 ligase domains. The resulting BioPROTACs used the scFv to recognize an exposed electrostatic‐loop epitope on misfolded SOD1, while the ligase module redirected the bound species toward intracellular clearance. Screening of seven scFvs identified BP2 as an effective binder, and further ligase comparison selected BP2–CHIP as the lead construct, later named MisfoldUbL. This degrader reduced aggregation‐prone SOD1 variants and showed preferential binding to SOD1A4V over wild‐type SOD1. MisfoldUbL mainly engaged ubiquitin‐proteasome degradation, with lysosomal clearance also contributing to aggregate reduction. In SOD1G93A mice, neuronal expression of MisfoldUbL delayed disease progression, reduced insoluble SOD1 in the brain, and preserved motor neurons and neuromuscular junction innervation. This work shows that scFv‐based TACs are not limited to extracellular target routing. When expressed intracellularly, scFvs can act as conformational filters that distinguish pathological protein states from native proteins.

A relative use of scFv is observed in the LIPTAC‐based degrader–drug conjugate study by Zhao et al. [60]. A hybrid bispecific format comprising an scFv arm and a Fab arm was developed to avoid heavy‐ and light‐chain mispairing during protein expression. The scFv format solves this mispairing problem by covalently linking VH and VL into a single polypeptide, so the binding arm no longer requires an independently expressed light chain. As a result, only the Fab arm still depends on the pairing of heavy chain and light chain, which further reduces product heterogeneity and makes the bispecific degrader well‐defined. This asymmetric Fab and scFv design made the construct easier to assemble and allowed the authors to test different spatial arrangements. In the EGFR‐targeting LIPTAC, the best‐performing format placed the LDLR binder 142F1 as the Fab arm and cetuximab as the anti‐EGFR scFv. This construct induced efficient LDLR‐dependent EGFR degradation and supported lysosomal payload release after drug conjugation.

A further extension of scFv‐based TAC design is exemplified by FolTAC‐dual [80], a folate receptor α‐targeting degrader built from antibody‐derived binding modules rather than full‐length IgG. On this platform, scFv serves as a compact, modular recognition unit that can be readily combined with additional binders to create dual‐target degraders. This feature is important because it allows scFv‐based constructs to go beyond single‐target depletion and support more complex receptor‐pair elimination. By co‐targeting EGFR/HER2 or PD‐L1/VISTA, the study showed that scFv‐containing degraders can be engineered into flexible multicomponent formats for simultaneous degradation of membrane proteins, while preserving the recombinant simplicity and architectural programmability of fragment‐based systems.

### 3.5 Nanobody‐Based TAC

Nanobodies introduce different design logic into antibody‐associated TACs. Unlike full‐length IgG, nanobody‐based TACs consist of only a single heavy‐chain variable domain, yet they maintain a compact recognition unit [81, 82]. As we previously discussed, the smaller size of a nanobody (∼15 kD) would facilitate penetration and access to structurally constrained extracellular epitopes [83]. Because nanobodies are single‐domain proteins, they are well‐suited for fully encoded degrader formats in which the binder is genetically fused to trafficking signals and sorting motifs. Their compact format also makes multivalent and multispecific designs easier to build, which is useful when degradation efficiency depends on avidity or coordinated receptor engagement. Another practical advantage is that nanobody‐based degraders do not rely on Fc‐mediated functions. This reduces undesired interaction with Fcγ receptors and removes one layer of biological complexity from the design.

With nanobody involvement in TACs, Zhang et al. introduced the GlueBody Chimera (GlueTAC), a covalent nanobody‐based strategy for targeted degradation of the immune checkpoint PD‐L1 [84]. This first nanobody‐based platform addresses key limitations of full‐length antibody‐associated degraders, such as poor tissue permeability and dependency on the expression levels of specific lysosomal targeting receptors (LTRs) or E3 ligases. GlueTAC utilizes a nanobody site‐specifically engineered with a proximity‐reactive, uncanonical amino acid, fluorosulfate‐L‐tyrosine (FSY), named Gluebody. After target binding, this residue enables irreversible covalent capture of the antigen. To drive internal trafficking, the GlueBody is conjugated to a chimeric peptide containing a cell‐penetrating peptide (CPP) for enhanced internalization and a lysosomal‐sorting sequence (LSS) for direct lysosomal routing. In this way, target recognition and routing are built into a single compact construct. This receptor‐independent design produced strong PD‐L1 degradation, reaching about 89% efficiency, and showed better performance than the full‐length antibody atezolizumab in both T cell activation assays and in vivo tumor inhibition.

While GlueTAC established a signal‐driven mechanism for targeted degradation by nanobody‐based TACs, the work by Han et al. repurposed this design for intracellular antigen delivery [85], called intratumoural vaccination chimeras (iVAC). Rather than treating PD‐L1 degradation as a terminal event, it treats the degradative route as the start point for antigen processing and presentation. To do so, the authors built a next‐generation degrader, GlueBody2. Instead of using FSY as in previous GlueBody, GlueBody2 replaced it with a more reactive, high‐affinity variant that enhanced covalent coupling to PD‐L1, and then an antigen peptide, such as an OVA epitope, was linked to the PD‐L1 degrader. As a result, when the chimera enters tumor cells through PD‐L1‐targeted internalization, it delivers not only the degradation module but also the antigenic peptide. This process effectively transforms tumor cells into “APC‐like” entities that cross‐present exogenous antigens on MHC‐I molecules to resident CD8^+^ T cells. In vivo study showed that iVAC administration successfully remodeled the immunosuppressive tumor microenvironment into an immunostimulatory state. This targeted antigen processing robustly reactivated exhausted T cell populations, leading to potent tumor regression and generating durable, systemic anti‐tumor immunity that protected against subsequent tumor rechallenge.

Like GlueTAC and iVAC, iLYTAC by Zhang et al. uses nanobody‐based target recognition to convert surface protein binding into active lysosomal degradation, but it does so through direct genetic fusion rather than chemical conjugation [86]. Specifically, the authors built iLYTACs as fully recombinant proteins by fusing target‐binding modules to IGF2, a natural ligand of CI‐M6PR/IGF2R. This genetically encoded design allowed expression and purification in *E. coli*, avoiding the heterogeneity and synthetic burden associated with chemically assembled LYTACs. After binding both the target and CI‐M6PR, the chimera is internalized and trafficked to lysosomes for degradation. The platform was not limited to a single target class. iLYTACs induced degradation of membrane proteins such as EGFR and PD‐L1, as well as extracellular targets including GFP and fibrillar α‐synuclein. The authors further developed two formats: type‐I iLYTACs, in which IGF2 was fused directly to an affibody or nanobody, and type‐II iLYTACs, in which IGF2 was fused to an IgG‐binding Z domain to create a universal adaptor for off‐the‐shelf antibodies.

The small size and simple structure of nanobodies make them particularly suitable for local expression of genetically encoded degraders. While a fully recombinant format of genetically encoded LYTAC has been developed, Yang et al. pushed this idea in a more localized and cell‐centered direction by developing genetically encoded LYTACs, named GELYTACs [87]. Rather than expressing and purifying the degrader in vitro, they engineered primary human T cells to secrete GELYTACs directly, armoring the therapeutic cell with a local source of protein degradation. The GELYTAC itself remained compact and fully protein‐based, consisting of a nanobody or scFv fused to IGF2 through a flexible linker. A key feature of this work was that the authors did not simply reuse native IGF2 because the secreted degrader levels cannot be tightly controlled in vivo, they optimized the lysosome‐targeting module by yeast surface display and generated the G2 variant with E6R, F19L, and S39P mutations. This improved binding to IGF2R, lowered the EC50 for target internalization from 97 nM to 22 nM, and reduced oligomerization during secretion. Functionally, the platform was used in a sender–receiver setting, in which engineered T cells secreted GELYTACs that promoted the uptake and degradation of soluble immunoregulatory factors, such as TGF‐β, and shed IL‐6R from neighboring cells. The main feature of this work is therefore not only that it retained the genetically encoded nanobody‐based TACs architecture, but also that it converted that architecture into a cell‐delivered, microenvironment‐focused strategy for local protein clearance.

**FIGURE 10:**
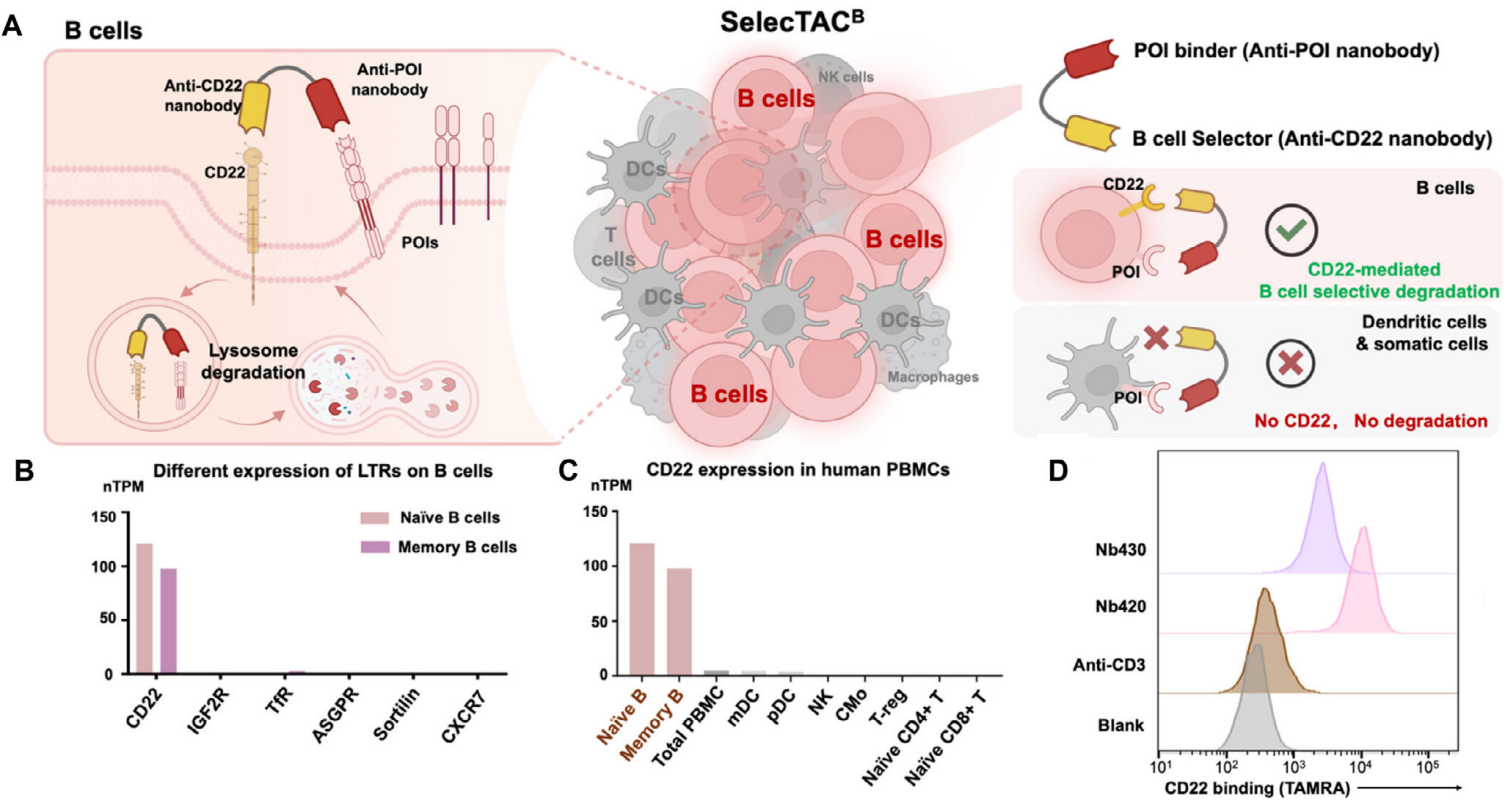
(A) Schematic of B cell‐selective targeting chimeras (SelecTAC^B^). (B) mRNA expression levels of different lysosome‐targeting receptors (LTRs) in B cells. (C) mRNA expression levels of CD22 in hPBMCs, illustrating CD22 highly expressed on naïve B cells and memory B cells. (D) Flow cytometry analysis indicating strong binding of two representative CD22 nanobodies (Nb420 and Nb430) to CD22 on Raji cells. Adapted with permission from Ref. [88]. Copyright 2025, American Chemical Society.

Building on the idea that engineered binders can actively direct membrane proteins into degradative trafficking routes, Yang et al. extended this strategy to cell‐type‐selective degradation by developing a nanobody‐based SelecTAC for B cells [88]. The authors chose CD22 as a B‐cell‐restricted lysosome‐targeting receptor, as most known lysosome‐targeting receptors are broadly expressed and cannot distinguish B cells from other immune populations. SelecTAC was built as a bispecific nanobody chimera, with one nanobody recognizing CD22 and the other recognizing the POI (Figure 10A). In this format, the anti‐CD22 arm provided both selectivity and internalization (Figure 10B), while the second arm defined the degradation target. The platform was first established with CD40 as a model target. In B cell lines, SelecTAC induced strong CD40 degradation at both the surface and whole‐cell levels. More importantly, the selectivity was maintained in human PBMCs (Figure 10C). Two nanobodies with strong binding ability to CD22 were selected as designed arms (Figure 10D). CD40 was efficiently reduced on primary B cells, while CD40 levels on T cells, NK cells, dendritic cells, and macrophages remained largely unchanged. This is the main strength of the work. It did not simply degrade a membrane protein. It degraded the same protein only on the intended immune cell type. CD40 loss reduced B cell activation in response to both soluble CD40L and CD40L‐expressing Jurkat cells, as evidenced by reduced upregulation of CD95 and CD86. Mechanistically, degradation was lysosome‐dependent and CD22‐dependent. The authors further expanded the platform to ICOSL and BCMA, showing that the same design could be adapted to additional B‐cell targets. Taken together, this work established a modular nanobody‐based strategy for cell‐selective membrane protein degradation on immune cells and introduced that a surface receptor can serve at once as a cell tag and as a lysosome‐trafficking module.

Nanobody makes E3 ligase‐recruiting TACs fully recombinant and modular. A nanobody can be designed as a bispecific form to serve as a toolbox for targeted protein degradation. Siepe et al. developed REULR as a modular nanobody‐based platform for cell‐surface protein degradation [89]. Rather than relying on lysosomal shuttling receptors or chemical conjugation, they built fully recombinant bispecific VHH molecules that recruit plasma membrane PA‐TM‐RING E3 ligases to non‐native surface targets. The study focused on five ligases with extracellular PA domains, namely RNF128, RNF130, RNF167, RNF43, and ZNRF3. By fusing ligase‐binding nanobodies to target‐binding modules, the authors forced proximity between these transmembrane E3 ligases and disease‐relevant receptors such as EGFR, EPOR, and PD‐1, which led to efficient degradation of target from the cell surface. The study also extended this strategy to the ligases themselves through “fratricide” REULRs. These constructs reduced the surface levels of RNF43 and ZNRF3. As a result, Frizzled receptors were stabilized on the cell surface, and canonical WNT reporter activity increased. This part of the work showed that REULR could do more than remove exogenous targets, it can also reshape endogenous signaling by changing the abundance of the recruited ligases.

This general design logic was later applied to a more defined cancer target in a compact bispecific nanobody format, as illustrated by the ROR1‐directed NbTAC reported by Zhao et al. NbTAC was used to eliminate ROR1, an oncofetal membrane receptor highly expressed in several cancers and associated with poor prognosis [90]. The design used a bispecific nanobody format to bring ROR1 into proximity with the membrane‐anchored E3 ligase RNF149. To build this system, the authors first identified nanobodies against the extracellular domains of RNF149 and ROR1 by phage display, then fused them into a series of bispecific constructs. Among these, N16‐R14 showed the best combination of stability and activity. In triple‐negative breast cancer cells, this NbTAC induced time‐ and dose‐dependent loss of ROR1, with substantial reduction after 48 h. Functionally, N16‐R14 also suppressed EdU incorporation and colony formation in multiple TNBC cell lines, whereas the individual nanobodies alone had little effect. This work is notable because it extends membrane protein degradation into a compact nanobody format and identifies RNF149 as a workable recruiter for extracellular target removal.

In 2026, Zhuang et al. developed a nanobody‐based PROTAC to induce degradation of the influenza nucleoprotein (NP), a highly conserved viral protein [91]. They fused NP‐binding nanobodies to the VHL E3 ligase domain and found that two constructs (VHL‐Nb135 and VHL‐Nb170) efficiently reduced NP levels across all 16 influenza A subtypes. This degradation strongly suppressed viral replication in cells infected with H1N1, H3N2, H5N1, H7N9, and H9N2 viruses. In mice, AAV‐delivered VHL‐Nb170 markedly lowered viral burden and protected most animals from lethal infection. This work shows that targeted degradation can be developed into a broad‐spectrum antiviral strategy.

### 3.6 Nanoparticle Mediated Antibody‐Associated TACs

Nanoparticles can be regarded as carriers for multiple antibodies, thereby converting the binding ability of antibodies into a TAC with degradability. By conjugation of antibodies onto nanoparticles, it provides a direct ability to deliver POI to certain receptors. In the meantime, the multivalent nature of antibodies would enhance their binding affinity to POI. Nanoparticles can also serve as a drug‐loading vehicle to accomplish the cooperation of LYTAC and cargo. Wang et al. designed an antibody‐enabled LYTAC by self‐assembling amphiphilic GalNAc glycopeptides (Lauryl‐P3GKS) into 200 nm nanospheres that bind membrane ASGPR and thus enter the endosomal and lysosomal route [57]. By using cetuximab and anti‐CD24 as model binders, antibody serves as an interchangeable recognition module that redirects the nanoparticle toward distinct membrane proteins for lysosomal depletion. Using an anti‐CD24 antibody as a model, the authors further showed that antibody‐mediated degradation has functional consequences beyond target removal, thereby weakening CD24–Siglec‐10 signaling and partially restoring macrophage phagocytosis in HCC. They further incorporated Gox cargo loading into LYTACs and demonstrated the potential to integrate antibody‐directed depletion with therapeutic cargo delivery via a single nanoparticle platform.

One of the most fascinating features of antibody‐associated LYTAC nanoparticles would be the interchangeable recognition module defined by the choice of antibody. In these systems, the antibody is no longer treated as a fixed part of a newly designed chimera. Instead, it serves as a replaceable targeting module mounted onto a common nanoparticle. This idea has precedent in earlier nanoparticle studies, in which an Fc‐specific anti‐IgG antibody was immobilized on the particle surface to create a general platform for mounting multivalent antibodies [92, 93]. That framework made it possible to load different antibodies onto the same nanoparticle with minimal loss of antigen binding, due to the oriented conjugation in the Fc region. In the context of targeted degradation, the implication is clearer. This shifts the design logic from building a new degrader for each target to reassigning specificity on a common platform.

**FIGURE 11:**
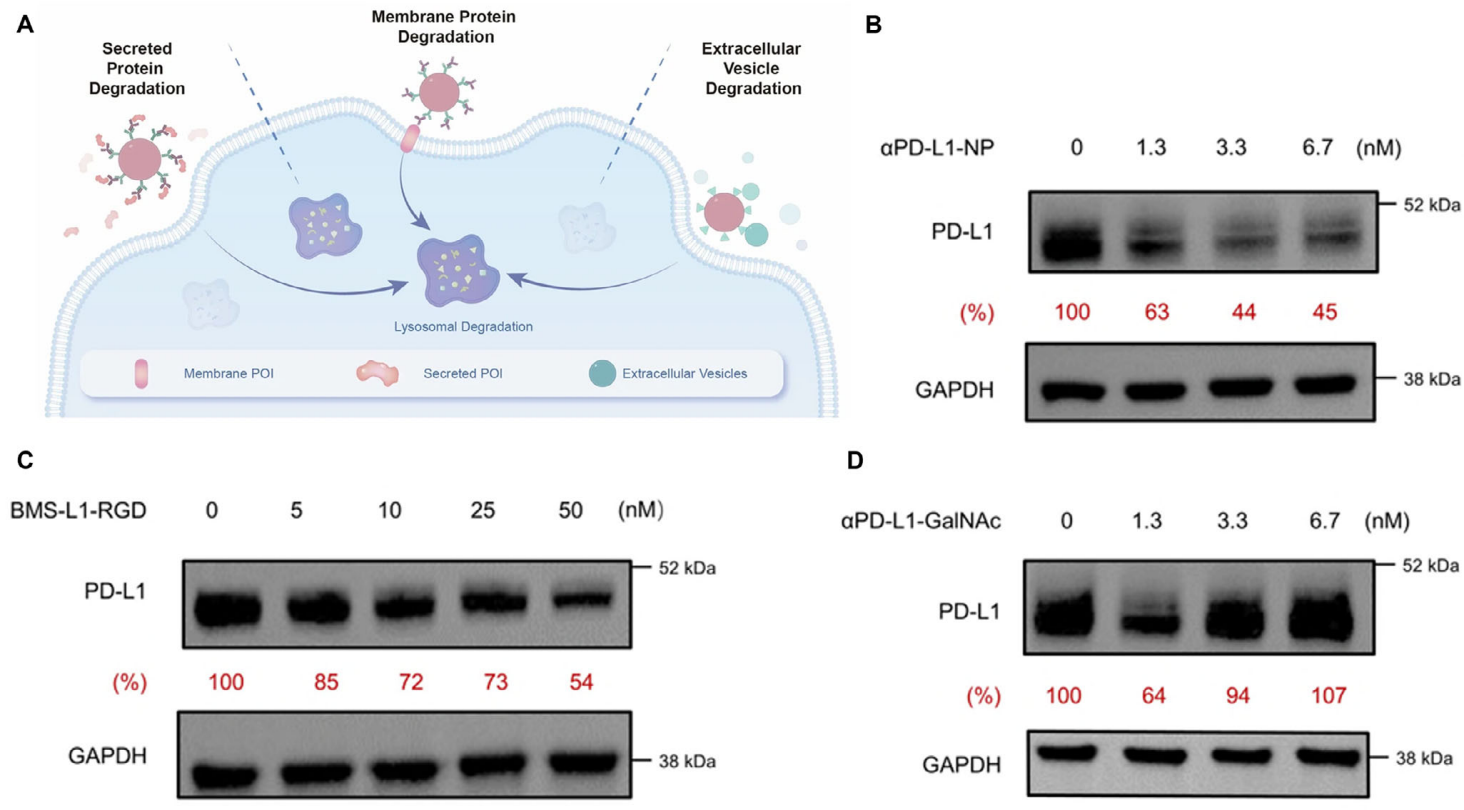
(A) Schematic of concept of MONOTAB degradation. Comparison of degradation ability between MONOTAB (B), small‐molecule PD‐L1 binder–RGD integrin degrader (C) and anti‐PD‐L1 antibody–GalNAc ASGPR‐targeting LYTAC (D). Adapted with permission. Ref. [49]. Copyright 2024, The Authors, published by Springer Nature.

Yao et al. carried that idea into extracellular targeted degradation as modified nanoparticles with targeting binders (MONOTAB) [49]. In this system, the nanoparticle is not simply a carrier for a preassembled bifunctional molecule. It becomes the degradative scaffold itself. The particle provides the lysosome‐trafficking route, while the mounted binder defines which target be captured and depleted (Figure 11A). Rather than rebuilding a new chimera for each POIs, it can preserve the same nanoparticle core and in the meantime change the antibody, which refer to as “plug and play.” The authors support this point by adapting the platform to distinct targets, including PD‐L1 (Figure 11B) and MMP2, and even extending it to extracellular vesicles. Compared with bifunctional PD‐L1 degraders such as BMS‐L1‐RGD (Figure 11C) and αPD‐L1‐GalNAc (Figure 11D), MONOTAB achieves stronger low‐nanomolar PD‐L1 degradation and avoids the high‐dose hook effect through its monofunctional nanoparticle‐based design.

Building on the idea that nanoparticles can serve as interchangeable antibody‐loading scaffolds, Xu et al. developed CD44‐Assisted Transcytosable chimeras (CATCHs) to address the physical challenge of solid tumor penetration [61]. CATCHs are built from a hyaluronan–polylysine nanoparticle decorated with Fc‐III‐4C peptides, which allows unmodified commercial antibodies to bind directly through the Fc region. Through binding between CD44 and hyaluronan on surface, CATCHs undergo CD44‐mediated endocytosis and are then partly routed through ER–Golgi‐dependent exocytosis, allowing the antibody‐loaded nanoparticles to transcytose across tumor cells while carrying bound target proteins. This feature gives CATCHs an advantage over conventional extracellular degraders, which often act mainly on cells exposed to the tumor surface. By moving through cell layers, CATCHs can penetrate to a deeper tumor region and continue target depletion there. Functionally, CATCHs rapidly depleted PD‐L1 and HER2 at low nanomolar antibody‐equivalent concentrations. PD‐L1‐targeted CATCHs reduced PD‐L1 across tumor tissue in vivo, increased CD8 T cell activity, and improved tumor control. This work therefore extends nanoparticle‐mediated TACs from modular antibody loading to active control of tissue penetration and deep‐tumor protein depletion.

Other perspectives would further refine the nanoparticle‐mediated antibody‐associated TACs. Beyond simply hosting a replaceable antibody, Li et al. showed that the particle itself can provide a lysosome‐directed trafficking route, even without relying on a canonical lysosome‐targeting receptor [94]. In their NanoTAC_PD‐L1_ system, antibody choice still defines the target, but degradation efficiency is now shaped by the nanoparticle's intrinsic uptake behavior and the biology of the treated cell. The paclitaxel‐induced PD‐L1 up‐regulation does not just work as a resistance mechanism of tumor cell, which commonly causes failure of traditional PD‐L1 antibody treatment. It is also turned into a condition that PD‐L1 accumulation and increasing affinity, which highly correlated with the efficiency of degradation, forming a strategy called “Meet‐Plot‐With‐Plot”. Once recognition and trafficking are partly uncoupled from fixed receptor‐ligand pairs, nanoparticle‐based degraders begin to look less like modified LYTAC mimics and more like autonomous degradation systems.

A similar perspective is provided by Song et al., which enhanced the endocytosis by coupling an anti‐PD‐L1 antibody with RGD peptides [95]. They conjugated aPD‐L1 and multivalent RGD peptides onto a poly(glutamic acid) nanoplatform, generating nanoparticles that can bind PD‐L1 and αvβ3 integrin simultaneously on the tumor cell surface. In this case, multivalent RGD strengthens αvβ3 engagement and improves lysosomal delivery of PD‐L1, thereby reducing receptor recycling and extending the effect beyond simple checkpoint blockade. This work provides another feature of nanoparticle mediated antibody ‐based TACs: degradation efficiency can also be dependent on optimized multivalent configuration and assisting modules like RGD peptide.

To construct functional modules, Cui et al. proposed an intelligent modular DNA LYTAC (IMTAC) nanodevice for assembling LYTAC [96]. By employing circular DNA origami, they designed modules with distinct functions, including lysosome‐targeting promoters, target‐binding ligands, and pH‐responsive switch elements, into a single nanodevice. This design allows precise control over module identity and relative arrangement, offering the degradation system more flexibility rather than a fixed molecular entity. The modular architecture further enables multitarget integration, as EGFR antibody and PD‐L1 antibody as binding units can be incorporated together to support collaborative degradation against various demands of tumor. In this sense, the paper advances LYTAC design from simple bifunctional conjugation to a more general bottom‐up assembly strategy for programmable, multifunctional degraders.

**FIGURE 12:**
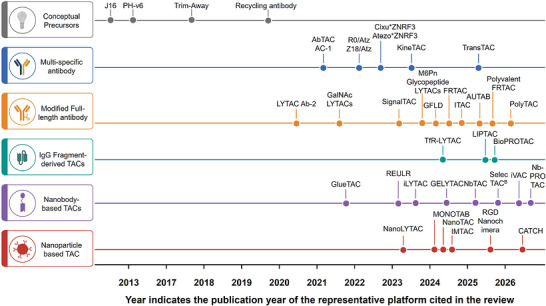
Timeline of representative AbTACs and related platforms grouped by scaffold architecture and arranged by the publication years of the platforms cited in this review. Figure created with BioRender.

Taken together, the platforms summarized that antibody‐associated TACs have expanded from a few early clearance‐inspired concepts into a broad and modular family of degraders (Table 1). Over the last decade, the rapid development of AbTACs has generated a diverse group of platforms with distinct scaffold formats. As shown in Figure 12, the timeline highlights the historical progression of the field from the early conceptual precursors of antibody‐mediated clearance to programmable antibody‐associated degraders. This evolution is not defined by a single mechanism, but by the increasing diversity of binder formats and trafficking strategies. Full‐length IgG scaffolds remain important because they are compatible with chemical conjugation, Fc engineering, and multispecific design. At the same time, scFv‐, nanobody‐, and nanoparticle‐enabled systems have introduced greater structural flexibility, greater recombinant simplicity, and tunable delivery behavior. Another clear trend is that the field is moving away from a strict dependence on classical lysosome‐trafficking receptors and toward a wider set of effectors, including membrane E3 ligases, transporter‐mediated uptake, autophagy‐linked routes, and modular particle scaffolds.

**TABLE 1:** Representative antibody binder‐based targeted degradation platforms classified by dominant molecular format and degradation strategy.

Category	Name	Binder format	Target	Reference
Conceptual precursors for antibody‐associated degradation	J16	Anti‐PCSK9	PCSK9	[67]
	PH‐v6	pH‐dependent anti‐human soluble IL‐6R IgG1 with engineered FcRn binding	human soluble IL‐6 receptor	[66]
	Trim‐Away	an intracellular target‐binding antibody and the cytosolic Fc receptor/E3 ligase TRIM21	GFP, Eg5, Rec8, huntingtin mutant, pericentrin, mTOR, IκBα, NLRP3	[65]
	PHD anti‐SEB recycling antibody	Engineered pH‐dependent variants of the anti‐SEB antibody 3E2	Staphylococcal enterotoxin B	[68]
	pH‐V3‐pI(A)‐N434A	pH‐dependent anti‐latent myostatin	myostatin	[70]
Multi‐specific antibody	AbTAC AC‐1	anti‐RNF43 Fab R3 + atezolizumab‐derived anti‐PD‐L1 arm	PD‐L1	[45]
	R0/Atz, Z18/Atz	anti‐RNF43 or anti‐ZNRF3 arms + anti‐POI arms	PD‐L1, EGFR	[72]
	Cixu*ZNRF3, Atezo*ZNRF3	anti‐ZNRF3 or anti‐RNF43 arms + anti‐POI arms	IGF1R, HER2, PD‐L1	[46]
	KineTAC	CXCL12 cytokine arm plus target‐binding Fab	PD‐L1, EGFR, HER2, VEGF, TNF‐α, PD‐1	[74]
	TransTAC	anti‐TfR1 scFv(H7) + POI binder	CAR, PD‐L1, EGFR, CD20	[47]
Modified Full‐length antibody	LYTAC Ab‐2	cetuximab‐M6Pn, atezolizumab‐M6Pn	EGFR, PD‐L1, CD71, ApoE4	[75]
	GalNAc‐LYTACs	ASGPR‐binding tri‐GalNAc ligand conjugated to antibody	EGFR, HER2, integrins	[42]
	SignalTAC	Antibody genetically fused to CI‐M6PR lysosomal sorting motif SFHDDSDEDLLHI	HER2, EGFR, PD‐L1, CD20, CD71	[48]
	M6Pn glycopeptide LYTACs	CI‐M6PR‐binding homogeneous M6Pn glycopeptide conjugated to antibodies	EGFR, CA9, c‐MET	[56]
	ITAC	Integrin‐binding cyclic RGD peptide cRGD conjugated to antibody	EGFR	[63]
	GFLD	Glut1‐binding glycooligomer conjugated to antibody	PD‐L1	[62]
	bisM6P‐antibody	Fc‐engineered NNAS anti‐TNF IgG	Soluble TNF	[43]
	FRTAC	Folate ligand conjugated to target antibody	EGFR, PD‐L1, CD47	[58]
	AUTAB	Antibody conjugated to PEI to induce autophagy	PD‐L1, CD73, integrin α5, EGFR	[76]
	Polyvalent FRTAC	Polyvalent folate‐conjugated antibody	EGFR, PD‐L1, TROP2, HER2	[59]
	PolyTAC	Target antibody or ligand conjugated to thiol‐reactive P10 polymer	PD‐L1, TROP2, VEGF, NA‐647	[77]
IgG Fragment‐derived TACs	TfR‐LYTAC	Genetically encoded TfR‐binding peptide fused to avelumab‐derived anti‐PD‐L1 scFv	PD‐L1	[78]
	LIPTAC	Bispecific antibody recruiting LDLR through anti‐LDLR Fab 142F1/142F6 plus POI binder	EGFR, PD‐L1, HER2	[60]
	BioPROTAC	Misfolded SOD1‐specific scFv fused to an E3 ligase domain,	Misfolded SOD1 variants	[79]
Nanobody‐based TACs	GlueTAC	Covalent anti‐PD‐L1 nanobody derived from KN035 with FSY	PD‐L1	[84]
	REULR	Bispecific VHH/nanobody format recruiting transmembrane E3 ligases RNF128, RNF130, RNF167, RNF43, ZNRF3	EGFR, EPOR, PD‐1	[89]
	iLYTAC	Genetically encoded IGF2‐fused binder	EGFR, PD‐L1, CD20, α‐synuclein	[86]
	GELYTAC	Genetically encoded binder fused to evolved IGF2	mCherry, TGF‐β, shed IL6R ectodomain	[87]
	NbTAC	Bispecific nanobody linking anti‐RNF149 nanobody N16 with anti‐ROR1 nanobody R14	ROR1	[90]
	SelecTAC^B^	Bispecific nanobody using anti‐CD22 nanobody as B‐cell‐selective lysosome‐targeting module plus anti‐POI nanobody	CD40, ICOSL	[88]
	iVAC	Covalent anti‐PD‐L1 nanobody/GlueBody conjugated with CPP and exogenous antigenic peptide	PD‐L1	[85]
	Nb‐PROTAC	influenza NP‐specific nanobody fused to VHL α‐domain	influenza A virus NP across H1–H16 subtypes	[91]
Nanoparticle based TAC	Nano‐LYTAC	ASGPR‐targeting GalNAc‐modified peptide nanosphere conjugated to antibody	CD24, EGFR	[57]
	MONOTAB	Streptavidin nanoparticle + biotinylated anti‐IgG Fc adaptor to recruit unmodified antibodies	PD‐L1, MMP2, extracellular vesicles	[49]
	CATCH	HA‐PLL nanoparticle chassis recruiting unmodified IgG through Fc‐III‐4C IgG‐binding peptide	PD‐L1, HER2	[61]
	NanoTAC	PLGA/DSPE‐PEG nanoparticle conjugated with anti‐PD‐L1 antibody	PD‐L1	[94]
	RGD nanochimera	Poly(glutamic acid) nanochimera bearing multivalent cRGD peptides with anti‐PD‐L1 antibody	PD‐L1	[95]
	IMTAC	Circular DNA origami nanodevice carrying pH‐responsive IGFIIR/CI‐M6PR aptamer promoter plus target ligands/aptamers	EGFR, PD‐L1	[96]

## 4 Challenges and Future in Translating Antibody‐Associated TACs

Despite the rapid expansion of antibody‐associated TAC formats, their successful design still depends on a limited set of practical constraints. In most cases, the key challenge is not simply whether an antibody can bind its target, but whether the resulting construct can recruit the appropriate routing system, maintain structural integrity, deliver the target to lysosomes, and remain effective and tolerable in vivo. These issues have now emerged as the main determinants of whether antibody‐associated TACs can move beyond proof of concept and become robust therapeutic platforms.

Receptor availability and tissue selectivity would be the first challenge, as many LTRs are not uniformly expressed across tissues and cell types. For current receptors, CI‐M6PR is attractive because it is broadly accessible, but this broad distribution weakens tissue selectivity and increases the risk of uptake at unwanted sites. ASGPR gives the opposite profile. While its strong hepatocyte bias is useful for liver‐directed degradation, it also confines the platform to a narrow anatomical setting. TfR1 enables efficient uptake, yet its robust recycling can counteract terminal lysosomal delivery if the target–degrader complex is repeatedly returned to the surface. Some receptors are even more restricted. Take CD22 as an example, it is useful precisely because it is largely limited to B cells, but this also makes it unsuitable as a general routing solution. Receptor choice is therefore not a secondary design variable. It determines which tissues are targetable, how selective the platform can be, and what biological liabilities are built into the degrader from the start. The species cross‐reactivity would be another consideration for the preclinical in vivo validation of degrader. Several antibodies and recruited receptors may differ between species used in preclinical studies and human, mouse models may not fully recapitulate the efficiency of AbTACs system in potential clinical applications. In the study of REULR, human and mouse cross‐reactive nanobodies were applied, which highlighted the importance of cross‐species compatibility in design and validation. Another strategy is to bypass the receptor dependence. Macropinocytosis‐mediated recyclable LYTACs (McR‐TACs) exploit receptor‐independent macropinocytosis to internalize membrane and extracellular proteins [97], enabling lysosomal degradation across diverse cell types while allowing the degrader to be recycled for repeated rounds of target capture.

Early antibody‐associated TACs often relied on random lysine conjugation because it was operationally simple and compatible with existing IgG scaffolds. Due to the broad application of lysine conjugation in AbTACs, product heterogeneity and conjugation control would also rise concerns. Broad lysine labeling creates mixtures in which ligand number and position vary across batches. As heterogeneity emerges, it becomes difficult to connect molecular structure with degradation activity, pharmacokinetics, or safety evaluation. This is the reason why the field has moved toward site‐specific conjugation, Fc glycan engineering, and recombinant fusion formats. These strategies do not simply improve chemical neatness. They produce more defined architecture and reduce batch‐to‐batch variability. Recombinant fusion proteins go even further, because the final product is encoded rather than statistically assembled.

Another issue is the correlation between endocytosis and degradation. Evidence of target uptake or of surface signal loss is insufficient to prove that a productive lysosomal event has occurred. A target may be internalized and then recycled back to the membrane, as often occurs with traditional antibodies. It may be neutralized extracellularly or transiently masked at the surface. These distinctions are especially important for membrane proteins with strong intrinsic recycling programs. For this reason, future work must more rigorously establish lysosomal fate. It is no longer enough to show internalization or short‐term surface depletion. The central question is whether the target is truly routed into the lysosome and degraded there, rather than merely redistributed. Tissue penetration and in vivo delivery would also define the therapeutic ability of AbTACs. Full‐length IgG‐based formats benefit from high affinity, bivalency, and long circulation, but their size limits penetration into dense tumor tissue. This remains a familiar trade‐off in antibody pharmacology. Smaller binders, such as nanobodies or scFv‐format AbTACs, can diffuse more effectively and access sterically restricted sites, but they often pay for this with a shorter serum half‐life and faster systemic clearance. Nanoparticle‐based systems introduce different compromises and can increase avidity, multivalency, and modularity. The best design depends on which limitation is dominant in a given application

The last concern for AbTACs translation would be safety and off‐target degradation. Many of them perform important endogenous functions, thus repeated engagement by engineered degraders may perturb native trafficking biology. Broadly expressed receptors also increase the risk of bystander uptake in nontarget tissues. At the same time, immune activation can become an unwanted mechanism. For example, Fc‐mediated effector functions, nonspecific immune cell engagement, and unintended inflammatory signaling may complicate the interpretation of degrader activity and introduce toxicity not directly caused by target removal. These concerns are especially relevant in antibody‐associated systems, because the scaffold carries biological functions beyond binding alone.

Small‐molecule TACs and AbTACs differ substantially in their administration routes and clinical development. Many small‐molecule TACs are designed for oral administration, which would improve convenience and enable repeated dosing. Intraperitoneal administration is also used in preclinical study for small molecule compounds with limited solubility. While AbTACs generally would apply systemic administration like intravenous injection, which benefited from their advanced circulation and bioavailability. With well‐established clinical experience with antibody therapeutics, the long serum half‐life of antibody‐based platforms may support less frequent dosing and sustained target engagement. AbTACs can also benefit from the antibody modification technologies to further improve its clinical performance.

As extracellular TPD developed, AbTACs are rapidly emerging as major contributors in this field. They are no longer just tools for pulling surface proteins into degradation pathways; they are becoming more programmable and functionally diverse. From an architectural perspective, the development of “plug‐and‐play” nanoparticle scaffolds has decoupled target recognition from lysosomal trafficking, enabling the rapid assembly of modular degraders. Chemically, more precise engineering strategies, including site‐specific conjugation and Fc glycan redesign, such as the NNAS switch, are improving structural definition and reducing heterogeneity. These chimeras now transcend localized target depletion to actively reprogram cellular immunity. Notably, the vaccination chimeras iVACs conceptually repurpose the degradative route as a starting point for exogenous antigen processing, effectively transforming targeted cancer cells into antigen‐presenting‐like entities. Taken together, these advances show that AbTACs are no longer simply surface protein degraders. They are becoming multifunctional systems for remodeling the tumor microenvironment with greater spatial, chemical, and biological control.

## Funding

This work was supported, in part, by METAVIVOR Foundation Early Career Research Grant Award to Q.H., American Cancer Society Research Scholar Grant RSG‐23‐1140821‐01‐ET (Q.H.), the National Institute of Biomedical Imaging and Bioengineering 1R01EB035992 (Q.H.), the National Cancer Institute 1R01CA288851 (Q.H.), Lachman Institute Research Funds, and Joseph R. and Bonna A. Robinson Chair fund, the University of Wisconsin Carbone Cancer Center Research Collaborative and the Pancreas Cancer Task Force (Q.H.), and the start‐up package from the University of Wisconsin‐Madison (Q.H.).

## Conflicts of Interest

The authors declare no conflicts of interest.

## Data Availability

Data sharing not applicable to this article as no datasets were generated or analysed during the current study.
